# Bioinformatic discovery of type 11 secretion system (T11SS) cargo across the Proteobacteria

**DOI:** 10.1099/mgen.0.001406

**Published:** 2025-05-21

**Authors:** Alex S. Grossman, Nicholas C. Mucci, Sarah J. Kauffman, Jahirul Rafi, Heidi Goodrich-Blair

**Affiliations:** 1 Department of Microbiology, University of Tennessee, Knoxville, TN 37996-0845, USA

**Keywords:** cargo detection, co-occurrence, DUF1194, host association, plasmin-sensitive protein, T11SS

## Abstract

Type 11 secretion systems (T11SS) are broadly distributed amongst *Proteobacteria*, with more than 3,000 T11SS family outer membrane proteins (OMPs) comprising ten major sequence similarity network clusters. Of these, only seven, all from animal-associated cluster 1, have been experimentally verified as secretins of cargo, including adhesins, haemophores and metal-binding proteins. To identify novel cargo of a more diverse set of T11SS, we identified gene families co-occurring in gene neighbourhoods with either cluster 1 or marine microbe-associated cluster 3 T11SS OMP genes. We developed bioinformatic controls to ensure that perceived co-occurrences are specific to T11SS, and not general to OMPs. We found that both cluster 1 and cluster 3 T11SS OMPs frequently co-occur with single-carbon metabolism and nucleotide synthesis pathways, but that only cluster 1 T11SS OMPs had significant co-occurrence with metal and haem pathways, as well as with mobile genetic islands, potentially indicating the diversified function of this cluster. Cluster 1 T11SS co-occurrences included 2,556 predicted cargo proteins, unified by the presence of a C-terminal *β*-barrel domain, which fall into 141 predicted UniRef50 clusters and approximately ten different architectures: four similar to known cargo and six uncharacterized types. We experimentally demonstrate T11SS-dependent secretion of an uncharacterized cargo type with homology to plasmin-sensitive protein. Unexpectedly, genes encoding marine cluster 3 T11SS OMPs only rarely co-occurred with the C-terminal *β*-barrel domain and instead frequently co-occurred with DUF1194-containing genes. Overall, our results show that with sufficiently large-scale and controlled genomic data, T11SS-dependent cargo proteins can be accurately predicted.

**Impact Statement**

We describe a novel method for controlling genomic neighbourhood analyses and use this controlled co-occurrence technique to examine two distinct clusters of genes from the recently described type 11 secretion system (T11SS): cluster 1, predominantly encoded by host-associated microbes, and cluster 3, encoded by marine microbes. We found that both clusters of predicted T11SS frequently co-occur with single-carbon metabolism and nucleotide synthesis pathways, but only the host-associated cluster co-occurred with iron uptake pathways. Using these datasets, we predicted 2,687 T11SS-dependent cargoes with approximately ten unique architectures, six of which have not previously been linked to T11SS. Finally, we validate our results by demonstrating T11SS-dependent secretion of a cargo protein with one of the novel architectures, plasmin-sensitive surface protein Pls from *Haemophilus parahaemolyticus*.

## Data Summary

This paper describes genomic co-occurrences of type 11 secretion system OMP sequences originally identified through sequence similarity networking in a previous publication [3] as constituents of similarity clusters 1 and 3. Accession IDs and assorted metadata for those clusters/networks are listed in Table S1 of that article: https://journals.asm.org/doi/suppl/10.1128/mbio.01956-21/suppl_file/mbio.01956-21-st001.xlsx. All other supporting data, accession IDs, code and protocols have been provided within this article or through supplementary data files. Additionally, all supplemental data are available on Figshare (https://doi.org/10.6084/m9.figshare.28514069.v2) [1].

## Introduction

Identifying protein function remains a difficult task in the postgenomic age. As of 2 December 2024, there were 5,366 domains of unknown function (DUF) families, which constitute around 22.6% of the database [2]. This means that roughly one-quarter of the Pfam database remains uninvestigated, limiting our understanding of the breadth of biological processes occurring on Earth. Functional characterization of proteins with DUF domains enables their annotation and removal from the DUF list, improving our datasets permanently. *In silico* approaches applied to protein domains are an important component of this process, as they can focus attention on the likely functions of a particular protein family [3]. Recently, a combination of *in silico* and experimental approaches has yielded insight into the DUF560 protein family of outer membrane proteins (OMPs), defining it as the bacterial type 11 secretion system (T11SS), represented by ten distinct sequence-level clusters [4]. T11SS is an OMP that translocates either soluble proteins or membrane-anchored lipoproteins from the periplasmic space to the extracellular space [4]. Presumably, cargo proteins are transported in an unfolded state, since some cargo proteins are dependent on Skp chaperones for secretion [5]. T11SS homologues are present amongst a diverse array of *Proteobacteria*, including human pathogens in the *Neisseria*, *Haemophilus* and *Moraxella* genera [6,8]. In the gamma-proteobacterium *Xenorhabdus nematophila*, a T11SS system encoded within the nematode intestinal localization (*nil*) is necessary for colonization of the mutualistic nematode host *Steinernema carpocapsae* [9]. In this system, the T11SS, NilB, regulates the surface exposure of a surface lipoprotein, nematode intestine localization protein (NilC), both of which are necessary for colonization. Other known lipidated T11SS-dependent cargoes include well-studied pathogenesis factors such as the metal uptake co-receptors transferrin-binding protein B (TbpB) and lactoferrin-binding protein B (LbpB), as well as factor H binding protein (fHbp), which binds an immune activation factor [6,8,10, 11]. These cargoes are composed of two types of domains, variable N-terminal effector domains and eight-stranded C-terminal *β*-barrel domains (such as TbpBBD and lipoprotein C). T11SS can transport unlipidated, soluble cargo proteins across the outer membrane. For example, T11SS-dependent haemophilin homologues have been characterized from *X. nematophila* (HrpC), *Acinetobacter baumannii* (HphA) and *Haemophilus haemolyticus* (Hpl) [4,10, 12, 13]. However, the majority of T11SS OMPs do not have a known or predicted cognate cargo. A sequence similarity network analysis suggested that experimentally characterized T11SS only captures a small portion of the functional diversity present in this family [4].

The availability and accessibility of sequencing data have skyrocketed since the first bacterial genome was sequenced in 1995, with ~315,000 prokaryotic genomes on RefSeq [14]. This tremendous repository of genomic sequence data allows computational biologists to probe these genomes for patterns in genetic co-occurrence, as measured through proximity to a gene of interest. Genes within a common functional pathway often cluster together within a genome. Such patterns can be useful when forming hypotheses about the function of uncharacterized proteins. Gene neighbourhood studies analyse the genomic regions upstream and downstream of any given gene of interest with the assumption that nearby genetic partners could illuminate the role(s) of the unknown gene [15].

However, one flaw in the current methodology used to derive co-occurrence lists is the lack of bioinformatic controls capable of removing non-specific co-occurring genes. For example, commonly inherited, deeply ancestral domains, such as transcription and translation machinery, often appear as the top result of a co-occurrence analysis but do not yield useful information about the specific query gene since their appearance is a coincidence of ubiquity [16]. Also, meaningful results can be obscured by co-occurrence based on structural or other common features present within the query protein, such as those that target the gene product to specific cellular locations, which are expected to interact commonly with the relevant protein trafficking machinery. Herein, we use computational approaches to elucidate potential functional roles played by T11SS-dependent cargo and predict novel cargo. Using these results, we then generated a co-expression system in *Escherichia coli* to experimentally demonstrate the secretion of one novel, bioinformatically predicted cargo family using the plasmin-sensitive protein (Pls) from *Haemophilus parahaemolyticus* and the type eleven Pls secretor (TepS).

## Methods

### Gene neighbourhood analysis

**Fig. 1.**
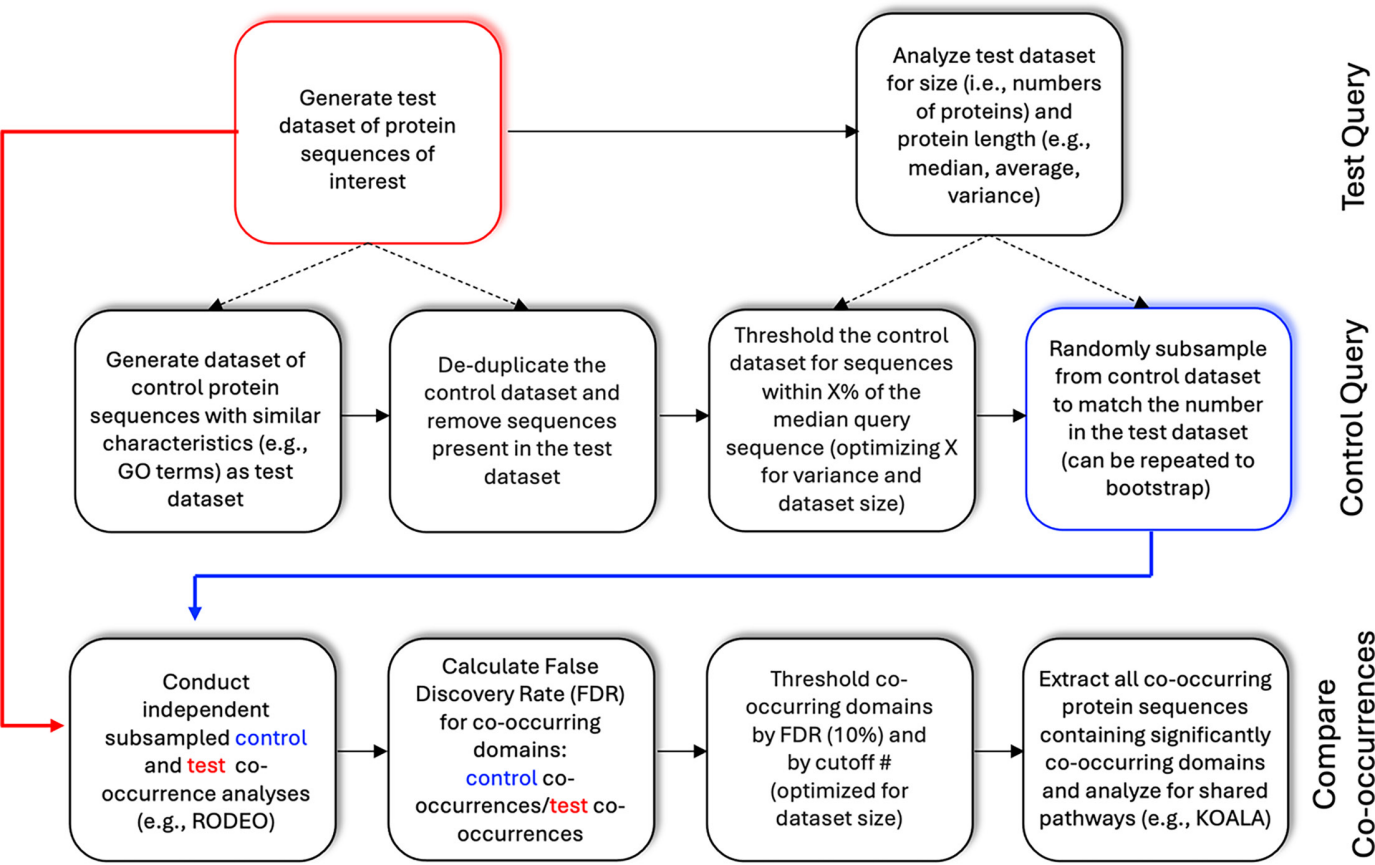
Design for a novel control technique for genomic neighbourhood co-occurrence analysis. Logic flowchart to control for non-specific co-occurrences from a genomic neighbourhood analysis. For the analyses presented here, the test dataset queries comprised two different clusters (1 and 3) of T11SS OMPs identified in a published network analysis [4]. Control datasets were developed for each cluster separately. The thresholds for the control datasets were set with X% determined as how many +/-aa resulted in the number of proteins that was close to, yet over, each test dataset.

The T11SS genome neighbourhoods (±6 genes around a T11SS gene) from cluster 1 (1,111 queries) and cluster 3 (145 queries) [4] were analysed. T11SS genome neighbourhoods were generated through Rapid ORF Description and Evaluation Online (RODEO) [17]. As described in the main text, a control was developed to identify and remove non-specific genes from amongst those flagged as co-inherited or syntenic with T11SS (Fig. 1). The primary control list was generated by extracting all GO-term OMPs from *Proteobacteria* and filtering for sequences similar in length to the median T11SS length (480–482 aa in length) and removing all DUF560-containing proteins. For both T11SS cluster analyses, a random subset of control sequences was chosen equal to the number of queries being analysed. Controlled protein sequences were downloaded from UniProt [18] in February 2021. Co-occurring protein domains were annotated and summed up to determine frequency amongst queries and controls. False discovery rate (FDR) was calculated by dividing the frequency amongst T11SS queries and control sequences. All domains with an FDR>0.1 were excluded from the analysis. For cluster 1, all domains with fewer than 25 occurrences were excluded. For cluster 3, all domains with fewer than five co-occurrences were excluded.

The Joint Genome Institute Integrated Microbial Genomes and Microbiomes platform (https://img.jgi.doe.gov/) [19,20] was used to examine genomic regions surrounding the T11SS OMP-encoding genes of select cluster 3 bacteria. Briefly, the ‘find genes’ function was used to identify T11SS OMP homologues from selected representatives of the *Roseobacteriaceae* and *Rhodobacteraceae*. The ‘gene neighborhoods’ function was then used to visualize the regions surrounding the T11SS OMP homologues and to identify syntenic loci (for an example, see File S3, Neighborhood Alignment tab). Predicted structures were downloaded as .pdb or .cif files from AlphaFold 2.0 [21,22] except in one instance (*Amylibacter cionae* CGMCC 1.15880 IEX10_RS12250) for which an AlphaFold 3.0 model [23] was used to predict the structure, and a .pdb file of the top-ranked prediction was used for Fig. 8(g) [21]. Average predicted local distance difference test values for each predicted structure are provided in File S1, available in the online version of this article (tab AlphaFold Predicted Structures). Using each .pdb file, structural motifs were highlighted using Protean 3D^®^, Version 17.4.3, DNASTAR, Madison, WI.

### Functional and structural analyses of co-occurring gene neighbourhood domains

Protein sequences that contain domains that co-occur with T11SS from the gene neighbourhood analysis (File S1, Cluster1FDR_Threshold tab) were obtained via UniProt [18]. KofamKOALA [24] and BlastKOALA [25] were used to assess function. KofamKOALA assigns Kyoto Encyclopedia of Genes and Genomes (KEGG) orthology to user sequence data by HMMER/HMMSEARCH against the KEGG database. BlastKOALA is similar but uses a blast search to assign KEGG orthology. Genes were matched to BRITE hierarchies to assess general cellular function and KEGG pathway to assess potential interactions.

To identify potential cargo proteins, all downloaded protein sequences were matched to UniRef50 clusters to compress potential homologues [26]. The reference sequences from these UniRef50 clusters were then subjected to a blast search against known T11SS cargo *β*-barrel and handle domains [NilC in *X. nematophila*, TbpB N-terminal and C-terminal in *Neisseria meningitidis*, haem receptor protein (HrpC) in *X. nematophila*, cobalt receptor protein (CrpC) in *Xenorhabdus cabanillasii*, haemophilin (Hpl) in *H. haemolyticus*, haemophilin (HphA) in *Acinetobacter baumanii*, fHbp in *Neisseria meningitidis*, and LbpB N-terminal and C-terminal in *N. meningitidis*] to search for homologues of T11SS cargo with a low stringency E-value filter of <0.05 in order to find even distantly related homologues. This generated a list of 141 potential cargo proteins from cluster 1 and a single potential cargo from cluster 3. Multiple sequence alignment was used to group the predicted cargo proteins into distinct architectures. Ten groups emerged: TbpB-like, LbpB-like, haemophilin-like 1 and 2, fHbp-like, Pls-like, highly disordered N-terminus 1 and 2, *Psychrobacter* surface protein and *Sphingomonas* surface protein. Structural data predictions for the nine architectures, which contained more than one sequence, were generated through PSIPRED [27] for the secondary structure and AlphaFold2 [21,22] for the tertiary structure.

### Co-expression of Pls and its cognate T11SS protein TepS

All cultures were grown in glucose minimal media supplemented with 1% Luria-Bertani broth. Plate-based cultures were grown on glucose minimal plates [28]. For plasmid-based expression, chemically competent *E. coli* strain BL21-DE3 (C43) was chosen for ease of transformation and their ability to non-toxically express membrane proteins [29,30]. Strains of *E. coli* were grown at 37 °C. Where appropriate, media were supplemented with ampicillin at a concentration of 150 µg ml^−1^. Protein expression was induced at the mid-log point of bacterial growth via the addition of IPTG at a concentration of 0.5 mM.

Using sequences from *H. parahaemolyticus* strain NCTC10794, GenScript synthesized genes for plasmin-sensitive surface protein (Pls-STO63610.1) with an attached C-terminal FLAG tag and its genomically associated T11SS OMP (TepS-STO63613.1) with an N-terminal 6xHis tag. Both genes were codon optimized for expression in *E. coli* BL21-DE3. GenScript cloned Pls-FLAG into multiple cloning site 1 of pETDuet-1 and 6xHis-TepS into multiple cloning site 2, resulting in expression plasmids pETDuet-1/Pls-FLAG and pETDuet-1/Pls-FLAG/6xHis-TepS. Expression plasmids were transformed into *E. coli* via electroporation. Strains were grown in a defined medium with 150 mg ml^−1^ ampicillin [31]. Bacteria were subcultured into 100 ml of broth at an initial OD at 600 nm (OD_600_) of 0.1, grown for 6 h at 37 °C to reach late logarithmic growth and induced with 0.5 mM IPTG for 1 h.

### Pls detection assays

Two approaches were taken to monitor the localization of Pls: immunoblotting (dot blot and Western) and immunofluorescence with flow cytometry. For the immuno-dot blot, cells were normalized to an OD_600_ of 1, rinsed 3×, lysed by sonication for 30 s at ~500 root mean square volts (V_rms_) and spotted in technical triplicate on nitrocellulose membranes to enumerate total cellular Pls. To monitor extracellular secretion of Pls, supernatant was collected from cultures after induction and filtered and sterilized to remove all cells. Supernatant samples were ultracentrifuged at 150,000 relative centrifugal force for 3 h to separate the soluble fraction from the insoluble component, composed of cell membrane components and membrane vesicles. Previous studies have detected both T11SS OMPs and cargo within membrane vesicles, necessitating their removal from supernatants [4,32]. The membrane vesicle fraction was suspended in a protein sample buffer. Seven hundred microlitres of each soluble supernatant fraction were precipitated via 10% TCA, and the resulting pellet was suspended in a protein sample buffer [33]. Samples were analysed by 10% SDS-PAGE and immunoblotting. All dot blots and Western blots were probed with rat anti-FLAG primary antibody and goat anti-rat secondary antibody conjugated to a 680 nm fluorophore. Intensities were recorded for FLAG reactive bands. For every supernatant sample, a band from the Coomassie blue-stained gel was used as a loading control to normalize the intensities of supernatant samples prior to analysis. Fold change of secretion was determined by dividing the amount of supernatant Pls detected in the Pls/TepS co-expression treatment by the amount detected in the Pls alone treatment. A Tukey’s honestly significant difference (HSD) test was used for comparing the fold change of secretion [34].

For immunofluorescence labelling and detection of Pls, overnight cultures of strains were washed thrice with PBS and resuspended in the defined medium at an OD_600_ of 0.05. Cultures were then grown to the mid-log phase, and one replicate culture of every strain was induced with IPTG at a final concentration of 0.5 mM, whilst the other culture was left uninduced. All strains were allowed to grow for an additional 1.5 h at 30 °C with shaking. Cells were blocked from non-specific binding using 3% BSA in PBS and incubated with a 1 : 300 dilution of DYKDDDDK Tag Recombinant Rabbit Monoclonal Antibody Alexa Fluor Plus 488 (Invitrogen) in 0.5% BSA in PBS. Cells were washed thrice in 0.5% BSA in PBS, fixed with 4% formaldehyde in PBS for 15 min and washed thrice with PBS. Cells were resuspended in PBS and then passaged through a Cytoflex (Cytek NL-3000), and 100,000 events were captured. Visualization and data analyses were performed in FlowJo (Beckton, Dickinson and Company, Franklin Lakes, NJ, USA). Flow cytometry analysis was performed three separate times, and for each experiment, three biological replicates were performed, resulting in a total of nine biological replicates. To monitor total Pls levels expressed, samples from cultures grown for the flow experiment were pelleted, washed with PBS and frozen at −20 for 24 h before being resuspended in PBS and lysed by sonication for 1 min total (5 s on, 10 s off, 600 V_rms_). After pelleting cellular debris, the protein concentrations of the cell lysate supernatants were determined using the Pierce 660 nm Protein Assay Reagent. All samples were normalized, and 9 µg of total protein for each treatment was separated by SDS-PAGE gel, followed by Western blot probing with primary anti-DYKDDDDK antibody (63703 BioLegend) at a 1 : 10,000 dilution and secondary IRDye 680RD Goat anti-Mouse (926–68070) at a 1 : 10,000 dilution. Blots were imaged using an Odyssey FC.

## Results

### Establishing a control for DUF gene neighbourhood co-occurrence analysis

Characterized T11SS-cargo pairs encoded by *Proteobacteria* typically have conserved roles in host-metal acquisition and immune evasion [4,8, 10, 12]. Only a small portion of predicted T11SS-cargo pairs has been experimentally characterized, leaving open the possibility that as-yet uncharacterized systems may be participating in diverse symbiotic and metabolic pathways. To gain insights into the potential breadth of biological processes in which T11SS-cargo pairs may function, we collected all T11SS sequences identified as part of the largest T11SS sequence similarity network cluster described in Grossman *et al*. [4] (cluster 1, containing 1,111 genes) and utilized the RODEO software to identify all protein domains encoded within their T11SS loci, herein defined as six ORFs upstream or downstream of the T11SS OMP query genes. Analysis of all loci resulted in a dataset of 1,504 domains, co-occurring between 2 and 928 times (File S1, Cluster1Co tab) [17]. To avoid spurious correlations, we chose to exclude any domains that co-occurred fewer than 25 times (~2.7% of the maximum frequency co-occurrence). This number was arrived at by generating a histogram of co-occurrence frequency and identifying the inflection point between the rare co-occurring sequences, which made up the bulk of the dataset (1,331 or 88.5%) and those domains whose co-occurrence frequency was greater than expected randomly (173 or 11.5%) (Fig. S1).

To ensure that the observed co-occurrences were specific to the T11SS protein family and not due to common features of genomic loci encoding OMPs, we developed and implemented a control process. Briefly, RODEO co-occurrence neighbourhoods were generated using randomly selected proteins with biophysical characteristics similar to our DUF560 query proteins such as protein length and outer membrane localization (GO term: outer membrane GO0019867) and then used as a point of comparison for gene neighbourhoods from T11SS genes. The control sequences obtained were limited to the *Proteobacteria* phylum, where T11SS predominantly occurs [4]. Due to a left-skewed distribution, the median length of all DUF560 domain-containing proteins in Pfam (accessed February 2021) was used to reduce bias from pseudogenes and gene fragments (Fig. S2) [2]. The median size of all predicted T11SS proteins was 481 aa, so we extracted all outer membrane ORFs between 480 and 482 aa in size. Sequences that appeared in the T11SS database were removed from the control database to ensure unbiased selection. A random subsample of this control database was taken equal to the number of sequences in the original query database (File S1, ControlQueries tab) (Fig. 1).

OMP sequences from the subsampled control dataset were then submitted as a query to the genome neighbourhood network function of RODEO using the same parameters as the T11SS query list. The output of this control co-occurrence analysis was used to generate an FDR for any given domain by dividing the frequency of co-occurrence with random OMPs by the frequency of co-occurrences with T11SS genes. We then gated the dataset for domains with an FDR less than 0.1 (10%) to exclude non-specific correlations, resulting in a final set of 51 significantly co-occurring domains. (File S1, Cluster1FDR_Threshold tab). The most common significant co-occurring domains alongside cluster 1 T11SS OMPs were other DUF560 proteins (928 out of 1,111), the TbpBBD *β*-barrel domain (503 out of 1,111), the TonB C-terminal domains (364 out of 1,111), haem oxygenase (137 out of 1,111) and DUF454 (118 out of 1,111). Next, all genes that co-occurred with a cluster 1 T11SS gene and possessed one of the identified domains were extracted, resulting in 3,405 significantly co-occurring genes (File S1, Cluster1SigGenes tab). These genes were submitted to KofamKOALA and BlastKOALA in parallel for annotation into molecular pathways and BRITE hierarchies [24,25].

### Functional assessment of T11SS co-occurring proteins identifies a role of iron uptake, protein export and single-carbon metabolism

**Fig. 2.**
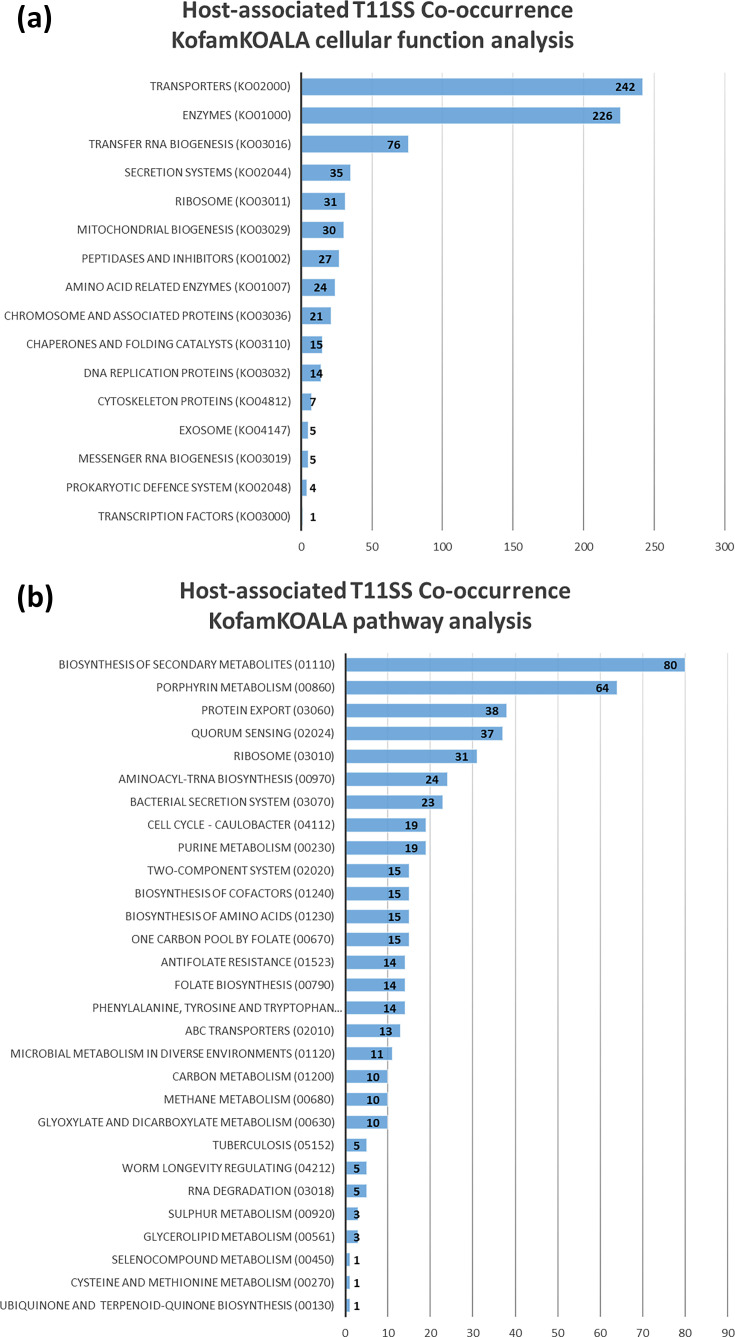
Host-associated T11SS genome neighbourhoods reveal conserved association with iron/haem uptake, export and one-carbon metabolism. KofamKOALA uses hidden Markov models to assign functions to query sequences and reveal shared pathways. Cellular functions (**a**) were estimated using BRITE hierarchies and, where possible, assigned to known pathways (**b**) to detect commonalities.

We examined the co-occurrence gene set for those with annotations in the curated KEGG Ortholog (KO) database [24,35]. A hidden Markov model search using KofamKOALA (HMMER/HMMSEARCH) was used to annotate the genes according to the KO database. The software found matches for 1,488 of the 3,405 significant co-occurring genes (File S2, Cluster1KofamBrite tab). When matched to cellular functions (BRITE hierarchies), the most common categories were transporters (periplasmic TonB, ExbD, signal peptidase II, TolA, etc.), enzymes (haem oxygenase, formate dehydrogenase, exopolyphosphatase, dihydrofolate reductase, etc.) and tRNA biogenesis (tRNA-modifying GTPase, tryptophanyl-tRNA synthase, ribonuclease P and aminoacyl tRNA synthase) (Fig. 2a). Additionally, cluster 1 T11SS genes were found to significantly co-occur with transposase genes. Of the 1,488 matched sequences, 259 had known pathway association, most commonly biosynthesis of secondary metabolites (haem oxygenase, cobalamin-dependent methionine synthase, vitamin K biosynthesis protein MenH, etc.), porphyrin metabolism (haem oxygenase) and protein export (signal peptidase II and YidC insertase) (Fig. 2b) (File S2, Cluster1KofamPath tab). The list of pathways also included aminoacyl tRNA biosynthesis (TyrS and TrpS). A high frequency of co-occurrence with transposases and tRNA synthesis genes is characteristic of pathogenicity islands, phage regions and other mobile genetic elements, since many mobile genetic elements have targeted insertion sites near universally conserved features, such as tRNA genes [36]. Co-occurrence with mobile genetic island signifiers supports our previous observation of horizontal acquisition of a T11SS in *X. nematophila* [4,9]. Finally, the list included several carbohydrate biosynthesis pathways (hsa00670, one carbon pool by folate; map00230, purine metabolism).

To further functionally annotate co-occurrences, we used blast alignment, rather than hidden Markov searching, to establish sequence similarity using BlastKOALA, which compares query sequences to the non-redundant KEGG dataset [25]. The software found matches for 1,387 of the 3,405 significantly co-occurring genes. Categorization into cellular functions (BRITE hierarchies) again revealed the largest categories to be enzymes, transporters and tRNA biogenesis (Fig. S3A) (File S2, Cluster1BlastBrite tab). BlastKOALA identified more co-occurring aa importers (arginine, lysine, tyrosine, tryptophan and serine transporters) in the dataset than KofamKOALA. Of the 1,387 identified genes, 260 had known pathways. This analysis identified similar pathways to those identified by KofamKOALA, including secondary metabolite biosynthesis, protein export, porphyrin metabolism and carbohydrate biosynthesis (Fig. S3B) (File S2, Cluster1BlastPath tab).

### Predicted structure analysis of putative cluster 1 T11SS cargo reveals variable N-termini and shared C-terminal domains

To bioinformatically identify potential cluster 1 T11SS-dependent cargo, we searched co-occurring protein sequences for homology to domains of experimentally demonstrated cargo [4,7, 12]. The 3,405 sequences significantly co-occurring with cluster 1 were first consolidated using UniProt ID mapper into 666 UniRef50 clusters with 50% or greater identity at an aa level (File S1, Cluster1Uniref tab) [26]. The reference sequences of these UniRef50 were next searched using BLASTp for sequence similarity with N-terminal domains and C-terminal *β*-barrel domains from experimentally verified T11SS cargo proteins: NilC, TbpB, LbpB, HrpC, CrpC, Hpl and fHbp, with a low stringency E-value cutoff of 0.05 [4,7, 8]. No matches were found for NilC or the N-terminal ligand-binding domain of fHbp, but all other queries matched at least one cluster. This analysis revealed 141 clusters of potential T11SS-dependent cargo proteins representing 2,656 ORFs across 1,048 species/taxa (File S1, Cluster1PutativeCargo tab). Each sequence was also annotated with SignalP 6 [37,38], which predicted 81 secreted lipoproteins, 33 secreted soluble proteins and 27 proteins with no predicted signal peptide (three of which were annotated as fragments). For our purposes, we defined a lipoprotein as any predicted to be membrane-anchored via tri-acylation of a C-terminal cysteine. Soluble proteins were defined as those predicted to have a Sec (or Tat) signal peptide and that lacked predicted transmembrane features. Overall, the fact that the majority of identified T11SS cargo candidates are predicted to have a signal peptide is consistent with published evidence that T11SS transports Sec-dependent lipoproteins and soluble proteins [4,7].

**Fig. 3.**
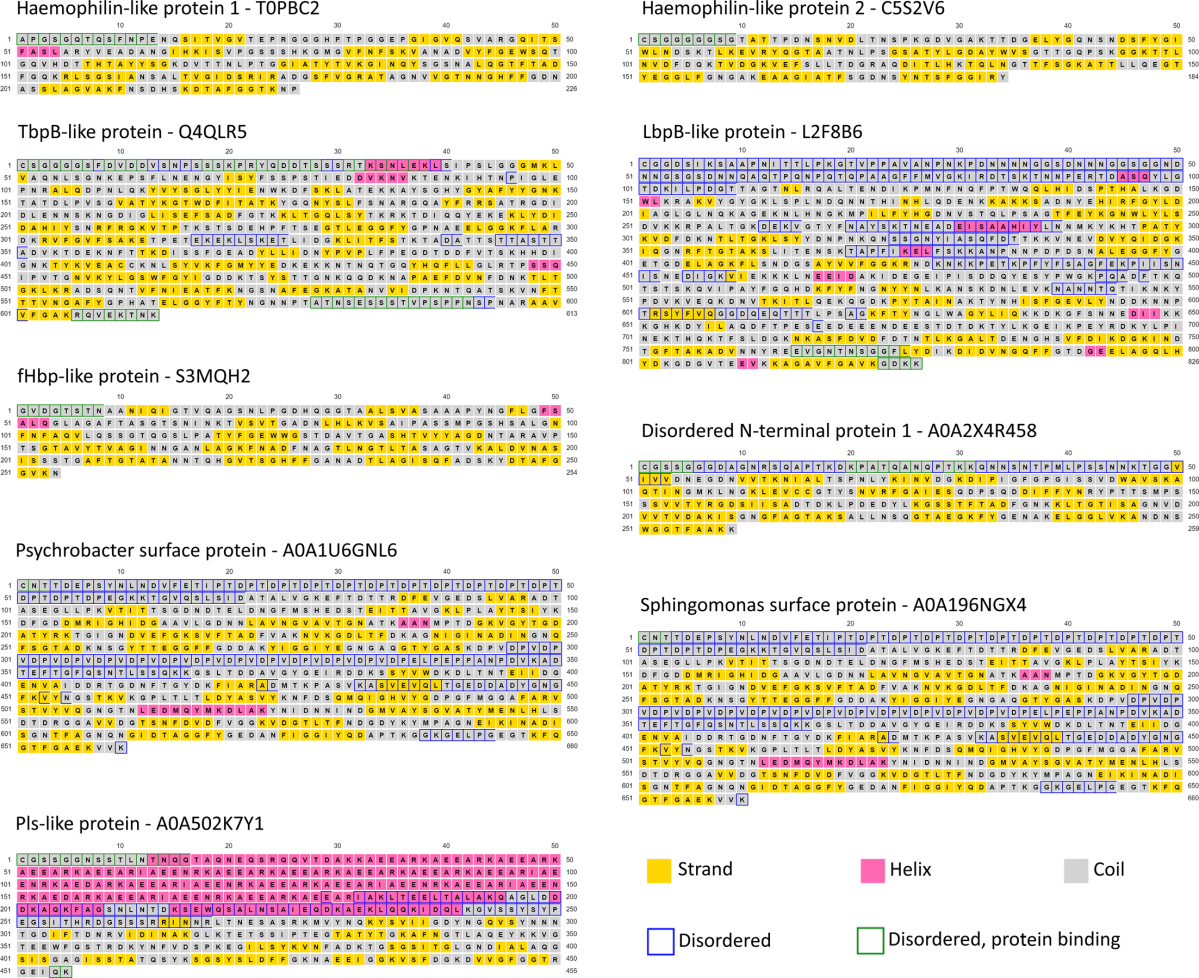
Secondary structure of predicted T11SS-dependent cargo reveals diverse and novel gene architectures and effector domains. PSIPRED 4 and DisoPred 3 use aa sequence to annotate secondary structures, highlighting a diversity of N-terminal domains with proline-rich repeats, intrinsically disordered regions, *α*-helical repeats and ligand-binding handles. For each of the detected cargo architectures, the representative sequence from the largest UniRef50 cluster was trimmed of its signal peptide to reflect a mature protein.

**Fig. 4.**
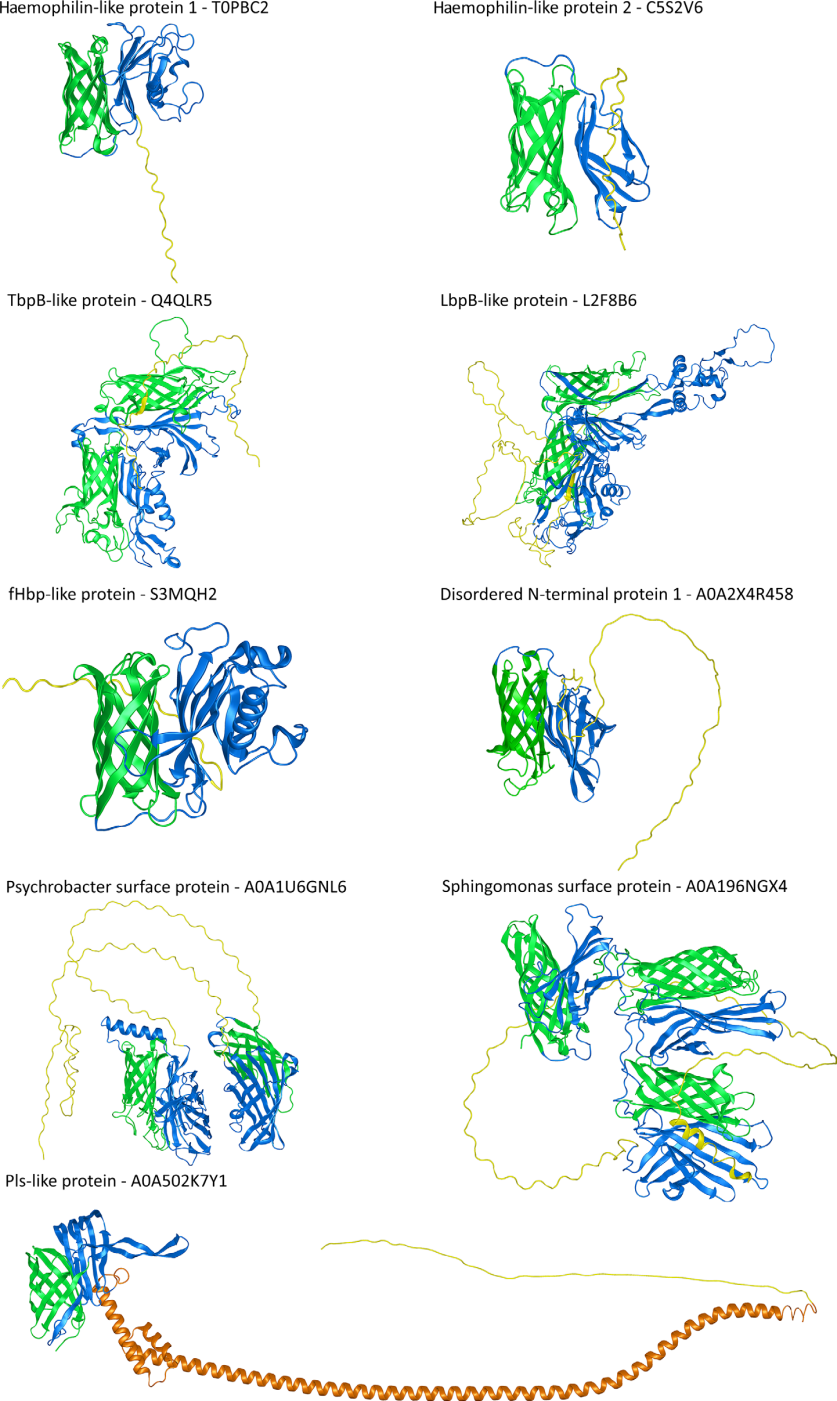
AlphaFold2 annotation of predicted T11SS-dependent cargo reveals conservation of C-terminal or centrally located *β*-barrel domains. For each of the detected cargo architectures, the representative sequence from the largest UniRef50 cluster was used to predict a representative structure. Green residues indicate C-terminal *β*-barrel domains. Blue residues indicate putative ligand-binding handle domains. Orange residues indicate glycosylation motif repeats within the Pls-like protein architecture. Yellow residues indicate regions predicted to be disordered.

We utilized multiple sequence alignment to separate the predicted cargo protein clusters into distinct, annotated architectures (secondary structures in Fig. 3, tertiary structures in Fig. 4 and File S1, Cluster1PutativeCargo tab). This analysis identified two distinctive groups of haemophilin-like proteins based on homology to known haemophilins (Hpl, HrpC and HsmA), the predominantly soluble group 1 (96 lipoproteins and 912 soluble) and the predominantly lipidated group 2 (65 lipoproteins and 7 soluble), a group of TbpB-like proteins (638 lipoproteins and 7 soluble), a group of proteins annotated as fHbp-like proteins by virtue of their lipoprotein C domain (54 lipoproteins and 285 soluble) and a group of lactoferrin-binding protein-like proteins (105 lipoproteins).

Several categories with no characterized representatives also emerged. Two lipoprotein architectures with an elongated, disordered N-terminal region occurred: disordered N-terminus 1 (196 lipoproteins and 8 soluble) and 2 (1 lipoprotein only). Another group was named Pls-like proteins (173 lipoproteins and 20 soluble) because of homology to the repeat-rich (EEARKA motif) *α*-helix from Pls surface glycoproteins in *Staphylococcus aureus*. Two phylogenetically-restricted architectures were observed with multiple TbpBBD domains, *Sphingomonas* surface proteins, which have an N-terminal sequence of proline-threonine repeats (18 lipoproteins and 12 soluble), and *Psychrobacter* surface proteins, which have an N-terminal sequence of ~12 proline-threonine-aspartic acid repeats (PTD motif) (55 lipoproteins and 4 soluble). Proline-threonine repeats occur in *Xanthomonas campestris* endoglucanase, where they provide elasticity and facilitate enzyme positioning around its substrate [39]. The PTD motif is found in a family of *Gracilibacteria* (BD1-5) surface proteins and in the eukaryotic microbe *Plasmodium falciparum* and is hypothesized to act as adhesins, facilitating interactions with hosts [40,41].

Using PSIPRED 4.0 [27] and AlphaFold2 [21], we predicted the secondary and tertiary structures of representative proteins from the largest UniRef50 cluster from each architecture with more than one representative (Figs3  4). All of the architectures were predicted to have at least one domain, generally at the C-terminus, composed of *β*-strands linked by disordered loops that form a C-terminal *β*-barrel domain similar to the TbpBBD domain or the lipoprotein C domain. The N-terminal domains of most cargo had *β*-strands predicted to form handle domains like those seen in haemophilin or TbpB [4,7]. When present, the disordered regions and long *α*-helical repeat regions preceded this handle domain.

**Fig. 5.**
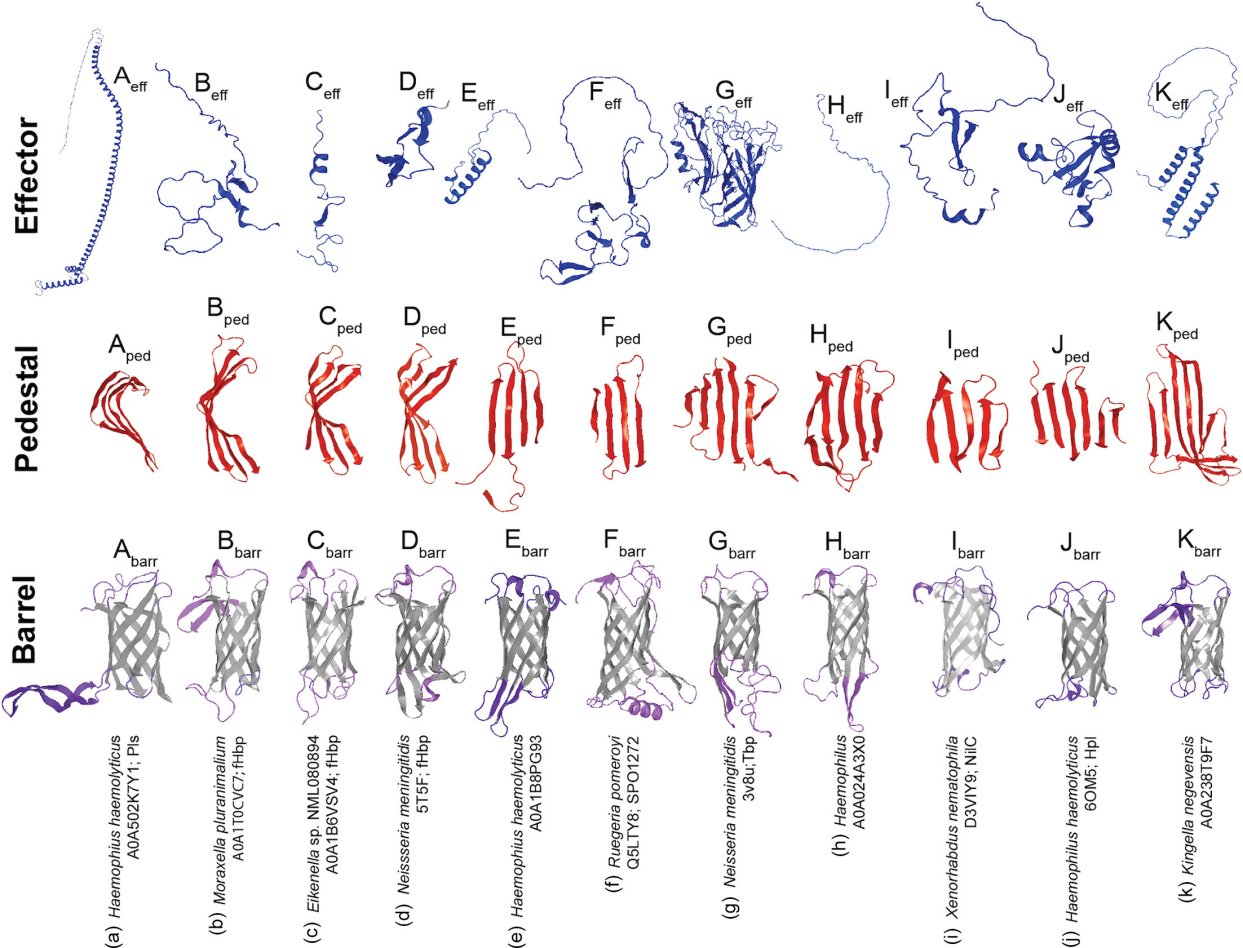
AlphaFold2 annotation of predicted T11SS-dependent cargo reveals three distinct domains ubiquitous to cargo. Solved (**d, g, j**) or predicted (**a, b, c, e, f, h, i, k**) structures from representative UniRefF50 cluster sequences of predicted (**a, b, d, e, f, h, k**) or known cargo (**d, g, i, j**). Each structure was separated into three sections representing common structural domains: N-terminal effector (blue), pedestal (red) and C-terminal *β*-barrel (grey barrel residues and purple loop residues). N-terminal effector domains varied in their predicted structural motifs and included disordered (**b, c, d, h, i**), *α*-helical (**a, e, k**), *β*-strand (**d, f, j**) and barrel (**g**). Pedestal domains comprised four-stranded groove (**a**), four-stranded ‘wing’ (**b–d**), four-stranded sheet (**e, f**), five-stranded sheet (**g–i**) or six-stranded/extended *β*-sheet (**j, k**). Some of these *β*-strands are donated by primary sequences from either the N-terminus or the C-terminus of the protein. Barrel domains exhibited variation in the presence of extended loops, helices or strands in predicted surface- or periplasmic-exposed regions, as indicated by purple coloration.

When examining secondary and tertiary structures in parallel, we noticed that the predicted cargo formed three distinctive structural regions: a common C-terminal eight-stranded *β*-barrel domain, a common central region formed by *β*-strands, which we have termed a ‘pedestal’ region based on its positioning as a platform for a variable N-terminal domain ‘effector’ domain, predicted to serve the functional role specific to each cargo protein type. Using solved crystal structures where available [6,10, 42], and AlphaFold2 or AlphaFold3 models for representative cargo proteins [21], we divided the predicted cargo structures into these three regions and categorized them by recurrent patterns (Fig. 5). The predicted central pedestal domains fell into six visibly distinguishable categories: four-stranded groove (Fig. 5a), wing (Fig. 5b–d) and sheet (Fig. 5e, f); five-stranded sheet (Fig. 5g–i); and six-stranded/extended sheets (Fig. 5j, k). None of the examined pedestal regions featured complex, variable loops, suggesting that they may play a structural role. In contrast, the C-terminal eight-stranded *β*-barrel core structures are embellished within each protein by diverse inter-strand loops including disordered regions, extended *β*-strands and *α*-helices that could be playing functional roles, as do the loops of the N-lobe TbpBBD domain of TbpB [43].

The N-terminal effector domains displayed a variety of structural motifs including disordered loops (Fig. 5b–d, h and i), *α*-helices (Fig. 5a, e and k), *β*-strands (Fig. 5d, f and j) and *β*-barrels (Fig. 5g). The examined effector regions ranged in size, with some barely extending beyond the pedestal domain and others including duplications of the eight-stranded *β*-barrel domain. The latter is typified by the solved structure of TbpB, which comprises two lobes, each containing the haemophilin handle-*β*-barrel pair architecture (Fig. 5g). The TbpB N-lobe pair comprises the effector region, whilst the C-lobe handle comprises the pedestal, and the C-lobe barrel is the putative T11SS targeting domain [42,43]. This domain duplication and functional specialization may also explain the architecture of *Sphingomonas* and *Psychrobacter* surface proteins that feature two to three copies of the handle-barrel pair (Fig. 4). However, the long, disordered loops present in those proteins likely generate a distinctive structural architecture relative to TbpB.

### Demonstrating T11SS-dependent secretion of plasmin-sensitive surface protein

To assess the accuracy of our bioinformatic predictions of T11SS cargo, we experimentally tested levels of secretion in *E. coli* of a representative novel cargo protein with or without its predicted cognate T11SS protein. Several of our predicted cargoes possess a large *α*-helix repeat region absent from the known T11SS cargo. We chose to test a representative of this group, the 525 aa Pls homologue (STO63610.1) and its genomically associated putative TepS (STO63613.1) from *H. parahaemolyticus* strain NCTC10794. The *H. parahaemolyticus* Pls *α*-helix repeat region shares between 49 and 62% identity with the repeat regions of surface glycoprotein Pls from *S. aureus* (WP_256928426, WP_256927786, WP_258808513, WP_256933640 and WP_257570445) that contributes to virulence by stimulating biofilm formation [44]. *Staphylococcus* and *H. parahaemolyticus* Pls differ in that homologues of the former are larger and lack the C-terminal *β*-barrel domain.

**Fig. 6.**
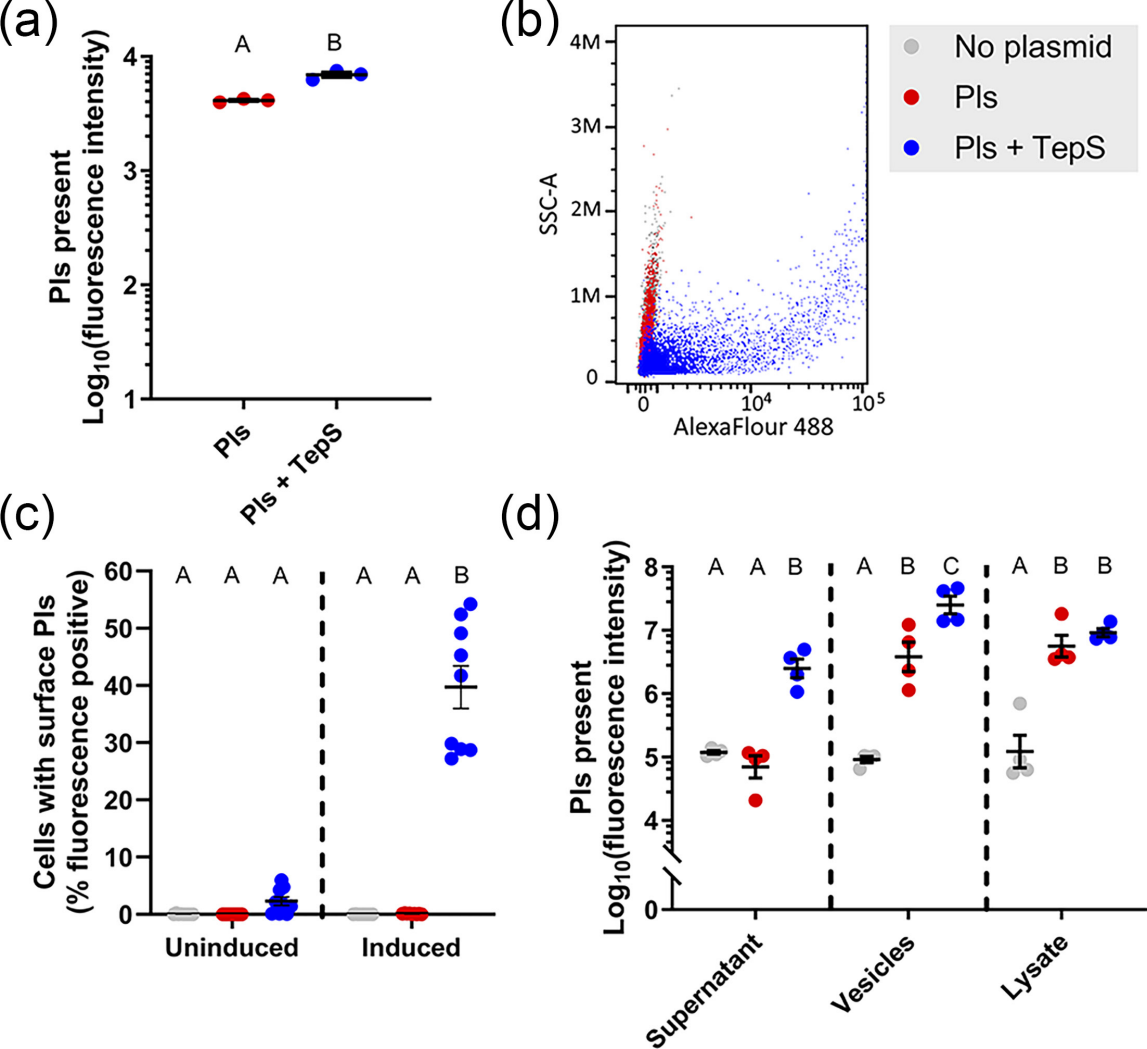
Localization of FLAG-tagged Pls in the absence and presence of its cognate T11SS secretor. *E. coli* cells expressing Pls-FLAG alone or Pls-FLAG alongside its cognate T11SS (TepS) were probed using Western blotting (lysates from flow cytometry samples, supernatants and extracellular vesicles), flow cytometry (cell surface exposure) and immuno-dot blots (lysates from supernatant collections) to see how the expression of the T11SS protein impacted protein localization. Strong expression of Pls was detected in lysates from both strains (**a, d**). A representative flow cytometry replicate (**b**) shows that surface-exposed Pls was only detectable on cells co-expressing TepS (**c**). For each biological replicate, 100,000 events were detected via flow cytometry. Supernatant secretion of Pls was also only detectable in the presence of TepS (**d**). The extracellular vesicle fraction collected via ultracentrifugation showed some Pls localization in the absence of TepS; however, that localization was significantly increased by TepS. One-way ANOVA with Tukey’s HSD statistical test was used to compare treatments in all experiments, and the letters above treatments indicate significance groups at a *P* value of 0.05. Error bars indicate sem.

To experimentally test for TepS-mediated secretion of *H. parahaemolyticus* Pls, we used the addition of a FLAG-tag epitope to allow Pls detection and expressed this protein with or without TepS using pETDuet-1-based plasmids in *E. coli* BL21 DE3 C43. Western blot analysis of lysed cells revealed that Pls was effectively expressed with or without TepS co-expression, though slightly more was present in/on cells expressing TepS, suggesting that surface exposure may increase the amount of protein a cell can contain or that TepS protects the protein from degradation (Figs 6a and S4AB). Flow cytometry was used to detect surface exposure of membrane-anchored lipoproteins. No Pls was detected on the surface of cells induced to express Pls alone; however, ~40% of cells had detectable surface Pls when expressing both Pls and TepS (Fig. 6b, c). Since Pls homologues can be cleaved spontaneously, or by proteases as in the case of *S. aureus* Pls [44,45], we used Western blot immunostaining on spent culture media (separated into soluble and vesicle fractions) to determine if Pls was being liberated from cell surfaces (Figs 6d and S4CD). Immuno-dot blots were performed on cell lysates after collecting supernatants to ensure that comparable amounts of Pls were being produced (Figs 6d and S4E) [44,45]. Pls was present in the supernatant, but only when co-expressed with the T11SS protein TepS, suggesting that *Haemophilus* Pls can be cleaved from the cell surface spontaneously [44,45]. Some Pls were located in extracellular vesicles independent of TepS expression, but this amount was higher in the presence of TepS (Figs 6 and S4). In Western immunoblots, FLAG-tagged Pls is visible as a single clear band in individual replicates but varied in apparent size between biological replicates from 82.3 to 121.3 kD with an average of 103 kD. This is almost twice the value predicted from the sequence alone (56.8 kD). This large and variable size may suggest that Pls is either being significantly slowed by glycan moieties as it travels through polyacrylamide, its passage is being affected by its extremely high concentration of charged aa or it exists as a dimer when expressed in *E. coli*.

### Bioinformatic analysis of cluster 3 T11SS proteins from marine environments reveals distinct co-occurrence patterns

To test the robustness of our analysis, we then utilized our co-occurrence technique to explore cluster 3 T11SS proteins, predominantly containing sequences from marine microbes. Cluster 3 was chosen for in-depth analysis because it is composed entirely of sequences from organisms that occupy aquatic/marine environments, for which no T11SS have been described in the literature and which may encode novel cargo. This cluster almost exclusively contains sequences from the families *Roseobacteriaceae* and *Rhodobacteraceae* [46], including both pelagic microbes and symbionts of algae (*Silicimonas algicola*) [47], molluscs (*Aliiroseovarius crassostreae*) [48], tunicates (*Ascidiaceihabitans donghaensis*) [49], echinoderms (*Sulfitobacter delicatus*) [50] and corals (*Roseivivax isoporae*) [51]. All genes from cluster 3 (145 sequences) were used as queries in the RODEO genome neighbourhood network function, using the same parameters as the cluster 1 analysis, resulting in 203 co-occurring domains with frequencies between 2 and 102 (File S1, Cluster3Co tab). Domains, which co-occurred fewer than five times, were excluded from the analysis (122 or ~60.0%) (Fig. S1C, D). Co-occurring domains were compared to another random subset of the non-specific control co-occurrence dataset to assign FDR values. All domains with an FDR greater than 0.1 were excluded from the analysis, resulting in 42 significantly co-occurring domains (File S1, Cluster3FDR_Threshold tab). The most common co-occurring domains were additional DUF560/T11SS domains (102 out of 145 loci), DUF1194 (102 out of 145 loci), glyoxalase domains (89 out of 145 loci) and thymidylate synthase/thymidylate synthase complementing protein (77 out of 145 loci). The TbpBBD domain passed the FDR threshold; however, relative to the analysis of cluster 1, it was exceptionally rare (8 out of 145 loci). All genes that co-occurred with a marine cluster 3 T11SS OMP and contained any of the significant co-occurring domains were extracted, resulting in 860 significant co-occurring genes, which we submitted to KofamKOALA and BlastKOALA for functional analysis [24,25].

**Fig. 7.**
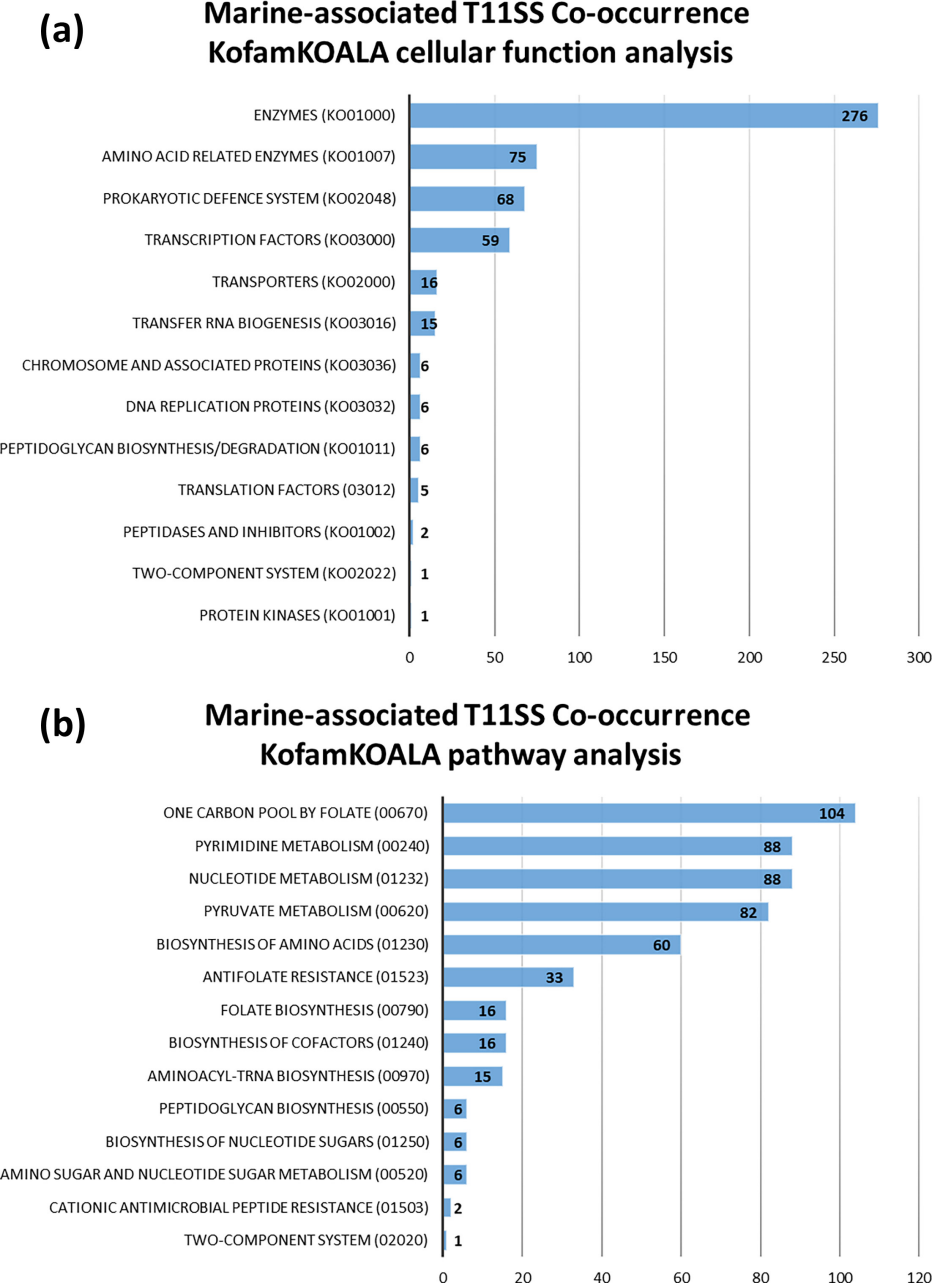
Marine T11SS genome neighbourhoods reveal an association with one-carbon metabolism, nucleotide metabolism and the glyoxalase-detoxification pathway. KofamKOALA uses hidden Markov models to assign functions to query sequences and reveal shared pathways. Cellular functions (**a**) were estimated using BRITE hierarchies and assigned, where possible, to known pathways (b).

KofamKOALA found matches for 595 of the 860 significant co-occurring genes (File S2, Cluster3KofamBrite tab). When matched to cellular functions (BRITE hierarchies), the most common categories were enzymes (lactoylglutathione lyase, thymidylate synthase complementing proteins, aspartate aminotransferase, etc.), prokaryotic defence systems (antitoxins CptB and HigA-1 and topoisomerase IV B) and transcription factors (cell division repressor DicA and cold shock protein) (Fig. 7a). Of the 595 matched sequences, 270 had known pathway association, the most common pathways being biosynthesis of one carbon pool by folate (dihydrofolate reductase and thymidylate synthase), pyrimidine/nucleotide metabolism (thymidylate synthase), pyruvate metabolism (lactoylglutathione lyase) and biosynthesis of aa (aspartate aminotransferase) (File S2, Cluster3KofamPath tab). Unlike cluster 1, transposases were not amongst the significantly co-occurring genes of cluster 3. However, aminoacyl-tRNA synthesis functions, which can be associated themselves with mobile genetic elements [36,52], do appear on the co-occurrence list, including alanyl-tRNA, prolyl-tRNA and threonyl-tRNA synthases (Fig. 7b). Parallel analysis with BlastKOALA identified 514 of the 860 significant co-occurring genes, of which 267 had known pathway association (File S2, Cluster3BlastBrite and Path tabs). BlastKOALA did not identify any additional cellular functions or pathways not already revealed by KofamKOALA (Fig. S5).

### Bioinformatic investigation of potential cluster 3 T11SS cargo

Initial attempts to identify new cargo proteins within the co-occurrence datasets from cluster 3 used the same methods used for cluster 1. UniRef50 clustering with ≥50% identity resulted in 218 homologous groups (File S1, Cluster3Uniref tab) that were assessed with blastp for sequence-level homology to experimentally characterized T11SS cargo domains. Only a few sets of homologues were identified, even when including all TbpBBD-domain-containing UniRef50 clusters. Homologues of six UniRef50 clusters, specific to the *Rhodobacteraceae*/*Roseobacteriaceae* families, resembled the haemophilin-like 1 architecture, and homologues from another UniRef50 resembled the disordered N-terminus 1 architecture noted previously (see Figs3  4). Collectively, these seven UniRef50 clusters encompass a total of only 31 ORFs across 27 species/taxa (File S1, Cluster3PutativeCargo tab). The ratio of predicted cargo per T11SS OMP was far lower in cluster 3 (0.21 cargo/input OMP) than it was in cluster 1 (2.39 cargo/input OMP). This dearth of co-occurring putative cargo proteins suggests that marine cluster 3 T11SS OMPs seldom co-occur with their cognate cargo proteins, utilize cargo proteins with below-threshold sequence similarity to those of cluster 1 T11SS or have evolved distinct functions and no longer have partner cargo proteins.

**Fig. 8.**
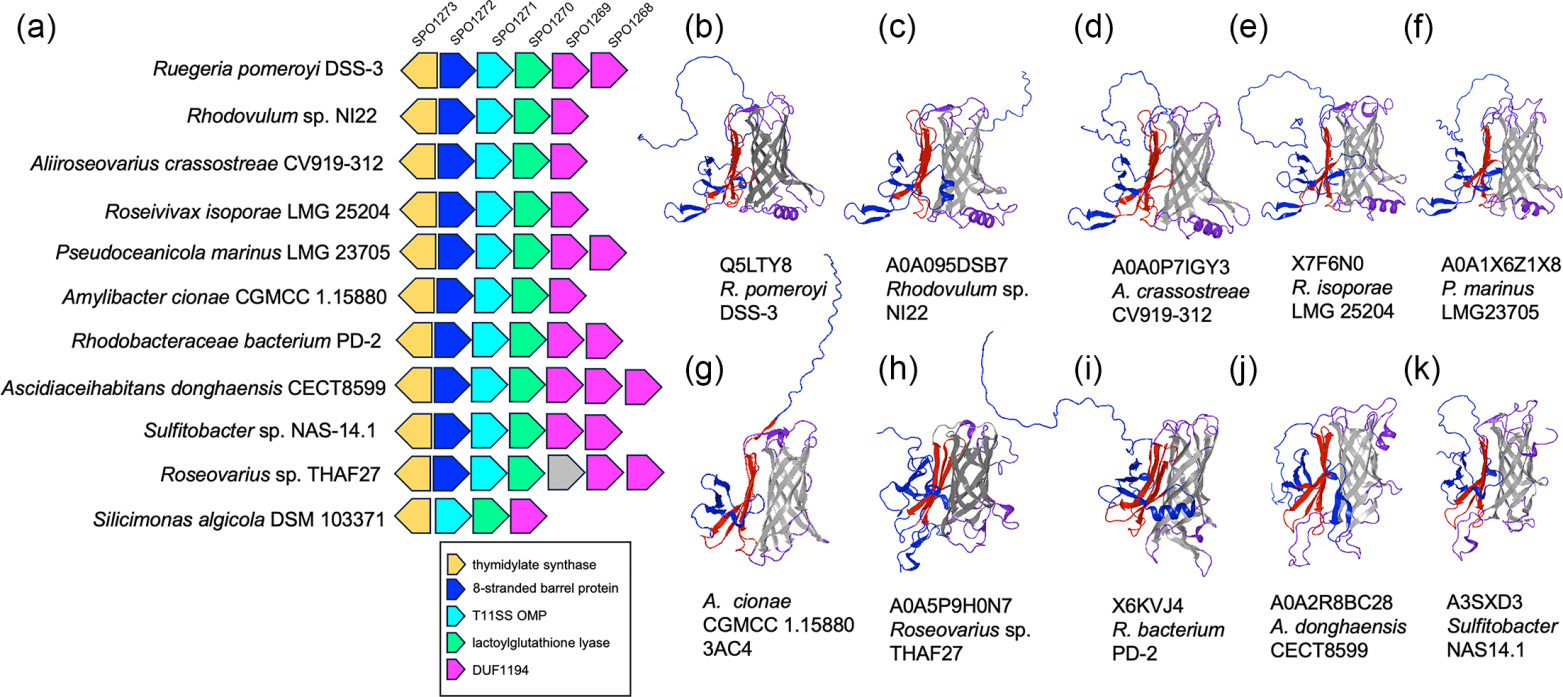
A conserved marine cluster 3 T11SS OMP locus encodes predicted cargo proteins. A schematic representation of select examples of a T11SS OMP-encoding, syntenic locus found with genomes of indicated strains of *Rhodobacteraceae* and *Roseobacteriaceae* (**a**). Each gene is represented by a block arrow (not to scale), and the *Ruegeria pomeroyi* locus tags of each homologue are indicated at the top. In all 11 genomes examined, the syntenic locus included genes predicted to encode a T11SS OMP (turquoise arrows) as well as the top cluster 3 co-occurring functions: flavin-dependent thymidylate synthase (gold arrows), lactoylglutathione lyase (neon green arrows) and one or more DUF1194/von Willebrand factor proteins (pink arrows). In all but one, the locus includes a putative T11SS cargo protein (light blue arrows), identified based on the presence of a predicted C-terminal eight-stranded *β*-barrel domain. Predicted AlphaFold 2.0 or AlphaFold 3.0 predicted structures of putative cargo proteins encoded within these loci are shown in (**b–k**). Colour coding is used to indicate the putative effector (dark blue), pedestal (red), barrel (grey) and surface or periplasmic loops (purple) of each protein. The top five appear to have similar structural motifs, including an *α*-helix in one of the *β*-barrel loops, antiparallel *β*-strands in the N-terminal domain (blue) and a four-stranded sheet pedestal domain (red). The remaining five structures show the overall domains of the N-terminal (blue), pedestal (red) and *β*-barrel (grey), but the N-terminal domains are predicted to have different structural motifs relative to the others examined.

To further examine this potential transition in function, we comparatively annotated a syntenic T11SS OMP-encoding locus in 11 representative *Rhodobacteraceae*/*Roseobacteriaceae* strains (File S3, Clus3SyntenicPairs tab). Each locus encodes all four of the top co-occurring domains from our co-occurrence analysis, including flavin-dependent thymidylate synthase, lactoylglutathione lyase and one or more DUF1194 (Fig. 8a and File S3, NeighborhoodAlignment tab). All but one (*Pseudooceanicola marinus* LMG23705) of the loci also encode a protein that is predicted to be a T11SS cargo lipoprotein (282–375 aa) based on the presence of an N-terminal SPII type signal sequence and a C-terminal eight-stranded *β*-barrel domain (Figs5f and 8bk) [21]. The presence of both cluster 1 and cluster 3 co-occurring domains indicates that this conserved locus is a hybrid of the two. It is tempting to speculate that this locus represents a transitional state in the divergence of these systems, with at least one analysed genome appearing to have lost the cluster 1 co-occurring, eight-stranded *β*-barrel domain.

We next examined the cluster 3 marine T11SS co-occurrence dataset to consider the possibility of alternative putative cargo types. In contrast to cluster 1 T11SS, co-occurrence with TbpBBD/lipoprotein C domains, TonB-dependent receptors and TonB domains is rare within the marine cluster 3 T11SS loci. Instead, the DUF1194 domain (PF06707) was highly prevalent (102 out of 145 queries) (File S4). Of the 102 DUF1194-containing sequences found in the cluster 3 co-occurrence list, 7 are predicted to be lipoproteins, 76 are predicted to be soluble Sec-secreted proteins and 19 have no detectable signal peptide according to SignalP 6 [37,38]. Many cluster 3 T11SS loci encoded two or three distinct DUF1194 genes in close proximity (e.g. Fig. 8), predicted to encode both lipidated and non-lipidated proteins (e.g. algal symbiont *Rhodobacteraceae* PD-2 ETA49263.2 and ETA49262.2, respectively) (File S4). The predicted RoseTTAFold structure [53] of the DUF1194 domain is a mixed *β*-strand/*α*-helix (Fig. 8). According to the Pfam database, the DUF1194 domain occurs in 31 distinct protein architectures with other domains, including the C-terminal autotransporter domain (PF03797), predicted to function in T5SS secretion; the CARDB domain (PF07705), which adopts a seven *β*-strand structures; DUF11 (PF01345), predicted to have an eight-stranded *β*-barrel structure; and PF18911, the known crystal structures of which also adopt a *β*-barrel (e.g., 1wgo and 2y72).

**Fig. 9.**
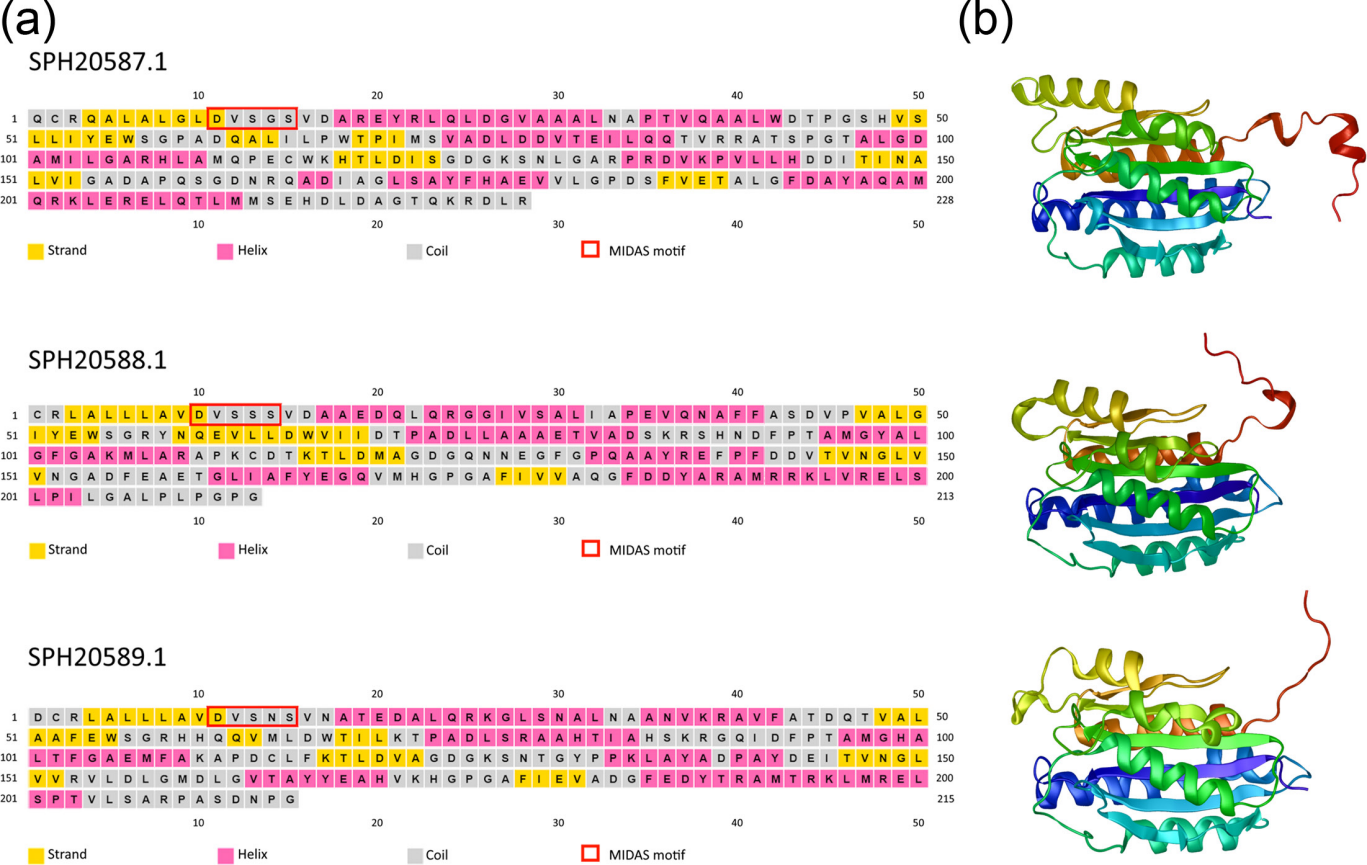
Representative structures of DUF1194 proteins located within a T11SS genomic neighbourhood in *A. donghaensis*. These three DUF1194 homologues are encoded adjacent to a T11SS OMP-encoding gene within a microbe isolated from a tunicate host. These proteins appear to be paralogues of each other based on sequence similarity. Signal peptides were predicted with SignalP 6 and trimmed from the sequences prior to submission to PSIPRED (**a**) and RoseTTAFold (**b**).

Of the co-occurring DUF1194 sequences, six are annotated as having homology to the von Willebrand factor type A (vWA) domain (PF00092). This homology is reflected in the structural similarity between these two domains, including the presence of a Rossmann fold, which may hint at similar molecular roles. vWA protein architectures can include divalent-cation-binding metal ion-dependent adhesion sites (MIDASs), with a characteristic DxSxS…T…D motif [54,55]. Almost all the DUF1194 proteins that co-occurred with DUF560 proteins contained at least one MIDAS motif (98 out of 102). To examine potential structural features of cluster 3 T11SS co-occurring DUF1194-containing homologues, we focused on three adjacently encoded DUF1194 genes from *A. donghaensis* (SPH20589/SPH20588/SPH20587) for structural analysis. PSIPRED 4.0 predicted that these proteins have at least five predominantly hydrophobic *β*-strands, all separated by *α*-helix regions in a structure that also appears to be a variation on the classical *α*/*β* Rossman fold (Fig. 9a). RoseTTAFold predictions of the tertiary structure of these DUF1194 proteins reveal globular proteins wherein six hydrophobic *β*-strands form a *β*-sheet at the core of the protein, which is protected by three *α*-helices per side. All three representatives had a MIDAS motif starting 10–11 residues after the predicted signal peptide. For each protein, the last 11–13 C-terminal residues appeared to be disordered, and their positions could not be estimated with a high degree of confidence (Fig. 9b).

## Discussion

The controlled bioinformatics analyses that we report here reinforce the idea that cluster 1 T11SS-dependent cargo has roles in metal uptake, single-carbon metabolism and nutrient provision. Further, our data extend the experimentally verified T11SS cargo to include *H. parahaemolyticus* Pls and lend credence to the concept that T11SS cargo can be bioinformatically predicted. The structural prediction that this cargo protein is a fusion between a functional Pls domain, typical of Gram-positive bacteria and a C-terminal eight-stranded *β*-barrel domain lends further evidence of the function of the barrel domain in directing cargo to the T11SS, and for the function of T11SS in facilitating transport across an outer membrane. However, our analysis of the more distantly related cluster 3 T11SS OMPs revealed that known barrel domains (TbpBBD and lipoprotein C) are rare amongst the significantly co-occurring genes identified, raising the intriguing possibility that novel structural domains may exist that mediate targeting to cluster 3 T11SS.

Our bioinformatic approach relied upon gene co-occurrence analysis, which uses genomic proximity as a proxy for coordinate regulation and potential interactions, since genes within a common functional pathway often cluster together within a genome. Whilst this is not a foolproof method, the use of large genomic datasets increases the power of co-occurrence analysis to identify potential co-interacting partners [56]. One of the methodological refinements that we developed was the use of *in silico* controls for genome neighbourhood co-occurrence to reduce false positives and noise in the output datasets of putative T11SS co-occurring protein families. For instance, activities associated with protein translation and RNA metabolism, which are essential cellular processes expected to be present in every cell, are not always useful co-occurrence signatures for elucidating T11SS function and components. Such general functions are not indicative of the specific functions of T11SS relative to any other OMP and can be considered a form of false negative, type II error; such co-occurrences are not biologically relevant but are not being excluded from the analysis because they do co-occur frequently with the query gene. However, the removal of such co-occurrences can also lead to false positives, type I error, because they can also result in the removal of some biologically relevant co-occurrences. For example, there could be protein families that are important functional partners of the query protein but are removed from the dataset if they were important for both query and control proteins. This type of error occurred in our analysis. For example, TonB-dependent receptors, with which many T11SS-dependent cargoes interact, were excluded from the cluster 1 T11SS analyses since these domains associate with other OMPs unrelated to T11SS. In a sense, this control narrows the focus from total co-occurrence to unique co-occurrence, and as such, it is best suited towards applications in which the researcher seeks to differentiate protein families. Additionally, the technique can be further adapted and refined, depending on the application. For instance, multiple non-specific gene neighbourhood controls could be generated by randomly subsampling the list of proteins that are biophysically similar to the query proteins, allowing for increased stringency and measures of variance.

The cluster 1 T11SS significantly co-occurring genes identified here are consistent with previous observations [4,7]. Iron uptake from the environment is a common function amongst experimentally verified T11SS-dependent cargo proteins, and this theme was extended within the cluster 1 T11SS co-occurrence dataset, which included haem oxygenases and haem transporters that facilitate iron uptake [57]. Similarly, the protein export genes identified as co-occurring with cluster 1 T11SS included TonB and ExbD, which are essential to energize the uptake of nutrients from known T11SS-dependent cargo [8,10, 58]. TolA is essential to bring periplasmic nutrients into the cytoplasm of the cell [59]. Signal peptidase II is essential for the generation of bacterial lipoproteins, which constitute a substantial percentage of T11SS-dependent cargo [60]. Finally, the observed co-occurrence with transposases and tRNA-synthases is consistent with the hypothesis that T11SS is regularly associated with mobile genetic islands and can be horizontally acquired and contribute to fitness within a host environment [36].

Significantly co-occurring pathways were commonly identified for both cluster 1 and cluster 3 T11SS and represent pathways not previously associated with T11SS. These included folate (vitamin B9)-related pathways, including folate biosynthesis, folate-dependent enzymes and one-carbon metabolism via folate. Folate, like haem, is an enzyme cofactor required for many diverse biological pathways. It is essential for the function of thymidylate synthase complementing protein, also known as flavin-dependent thymidylate synthase, in nucleotide biosynthesis [61]. This protein is the third most common cluster 3 marine T11SS co-occurring gene, suggesting coordinate regulation or inheritance. Since, unlike haem, folate does not incorporate a metallic ion, and most bacteria synthesize their folate instead of scavenging it from the environment [62], we do not favour the hypothesis that folate itself is acquired through a T11SS-dependent uptake mechanism. Instead, the close association of folate metabolism and T11SS may reflect the fact that folate biosynthesis, as well as iron-sulphur cluster biosynthesis, and methylotrophic metabolism by formate dehydrogenase (which also significantly co-occurred with T11SS) depend on the metal-bearing enzyme cofactors, cobalamin (vitamin B12) and molybdopterin cofactor (MoCo) [63,66]. Our data suggest that some T11SS OMPs may function in cobalamin or molybdopterin uptake pathways. Regardless, our data clearly demonstrate an association between T11SS and single-carbon metabolism and methyltropism.

Cluster 1 T11SS co-occurrences featured TonB energization or haem/iron uptake that were lacking from cluster 3 T11SS co-occurrences. In turn, the most frequent cluster 3 T11SS co-occurrences were DUF1194 and lactoylglutathione lyase (GloA), neither of which appeared in the cluster 1 T11SS co-occurrence datasets, but both of which have connections to metal ions. The function of DUF1194 is currently unknown, but it is predicted to fold into a globular protein with an *α*/*β* Rossman fold. Several of the DUF1194 proteins (6 out of 103) that we detected also had homology to a structurally similar *α*/*β* Rossman fold domain called vWA, which is most often associated with serum glycoproteins and large multimeric protein complexes in mammalian systems [67,68]. In *Myxococcus xanthus* bacteria, the vWA domain-containing surface lipoprotein CglB acts as a surface adhesin essential for gliding motility [69]. Upon binding a ligand, vWA domain-containing adhesins such as CglB can stabilize their bond via a conformational change induced by MIDAS motif allosteric binding of a divalent metal cation, often Mg^2+^ [55,69]. The presence of a MIDAS motif in the vast majority of DUF1194 identified within our dataset suggests that these proteins may also play a role in adhesion or may participate in metal homeostasis in previously unpredicted ways. Additionally, vWA domain-containing proteins are involved in microbial conflict sensor systems called MoxR-vWA-centric ternary systems, wherein a surface receptor protein receives an extracellular signal from a symbiotic species and then signals a response using intracellular vWA chaperones and MoxR ATPases [70,71]. Although we did not detect MoxR homologues in our analysis, this example raises the possibility that one role for T11SS OMPs may be to deliver sensor proteins to the cell surface that relay signals to DUF1194 partners. Other possibilities include that DUF1194 proteins themselves are a type of T11SS-dependent cargo or that they contribute to a multimeric protein complex that interacts with T11SS proteins. Lactoylglutathione lyase is an enzyme with a coordinated metal ion (typically nickel) and functions in the regeneration step of the glyoxalase-mediated aldehyde detoxification system [72]. It is possible that the nickel requirement of this enzyme reflects a role for cluster 3 T11SS in facilitating nickel uptake. Alternatively, this co-occurrence may indicate that cluster 3 T11SS is more generally participating in an aldehyde-producing process. Notably, aldehydes like methylglyoxal are exceptionally cytotoxic and can damage DNA and proteins, so the cluster 3 T11SS co-occurrence of nucleotide and aa biosynthesis may reflect a more general relationship between T11SS and the aldehyde repair response [72,73].

In addition to providing insights into functional pathways associated with T11SS, our analysis also allowed us to identify 148 homology groups of putative T11SS-dependent cargo, including those with domain architectures distinct from those found in known cargo. We verified that at least one of these, the plasmin-sensitive surface protein (Pls) from the Gram-negative organism *H. parahaemolyticus,* is a bona fide T11SS-dependent cargo protein, demonstrating the efficacy of our bioinformatics approach. Pls represents a novel N-terminal effector domain amongst T11SS cargo. This domain is homologous to glycosylated Pls proteins found on or spontaneously cleaved from the surfaces of Gram-positive organisms such as *S. aureus* and *Staphylococcus epidermidis* where they function in biofilm formation and modulating adhesion to host proteins [44,45]. Given that the function of Pls requires surface exposure, its presence in a Gram-negative organism is complicated by the existence of the outer membrane. In *H. parahaemolyticus*, this hurdle seems to have been overcome via fusion of the Pls repeat domain to the C-terminal eight-stranded *β*-barrel domain necessary for cluster 1 T11SS-dependent secretion across the outer membrane [4,7].

When co-expressed in *E. coli* with its T11SS partner, TepS, Pls predominantly localizes to cell surfaces and the supernatant. Our working model is that in the presence of the T11SS TepS, Pls is secreted and then cleaved, releasing it into the extracellular milieu. Given that our experiments were conducted in a protease-deficient strain of *E. coli*, it will be exciting in future studies to determine if TepS alters the topology of Pls in order to make it susceptible to proteolysis, or if TepS is directly responsible for Pls cleavage, which would be a novel function for T11SS. The possibility that Gram-negative Pls is secreted via T11SS and may be released from the cell surface in the process will be an important factor to consider in ongoing efforts to target Pls homologues for vaccine development, such as that being pursued for *Actinobacillus pleuropneumoniae* Pls (YP_001652736.1) [74]. Also, the Pls-like proteins have repeat-rich regions, and the possible glycosylation of *H. parahaemolyticus* Pls is supported by our observations of its aberrant mobility in SDS-PAGE. It is possible that other T11SS-dependent cargo, including lipoproteins, may also be glycosylated. If so, such glycoproteins tethered to the cell surface would display glycans as surface antigens that may modulate host-microbe recognition and immune evasion [75,77].

In summary, our findings have enhanced our understanding of potential effectors of the novel T11SS and expanded the list of experimentally verified T11SS-secreted cargo. We have also expanded the field of genomic co-occurrence analysis by establishing a protocol that can serve as a basis for more controlled and specific experimental designs. Exciting avenues of future research could focus on generating an automated pipeline for controlling co-occurrence analyses, determining if Pls is required for host colonization by *H. parahaemolyticus*, investigating if DUF1194 represents a novel T11SS cargo type and exploring the relationship between one-carbon metabolism and T11SS.

## Supplementary material

10.1099/mgen.0.001406 Uncited Supplementary Material 1.

10.1099/mgen.0.001406 Uncited Supplementary Material 2.

10.1099/mgen.0.001406 Uncited Supplementary Material 3.

10.1099/mgen.0.001406 Uncited Supplementary Material 4.

10.1099/mgen.0.001406 Uncited Supplementary Material 5.

## Abbreviations

CrpC: cobalt receptor protein
DUF: domains of unknown function
FDR: false discovery rate
fHbp: factor H binding protein
HrpC: haem receptor protein
HSD: honestly significant difference
KEGG: Kyoto Encyclopedia of Genes and Genomes
KO: KEGG Ortholog
LbpB: lactoferrin-binding protein B
MIDASs: metal ion-dependent adhesion sites
NilC: nematode intestine localization protein
OD_600_: OD at 600 nm
OMPs: outer membrane proteins
Pls: plasmin-sensitive protein
TbpB: transferrin-binding protein B
TepS: type eleven Pls secretor
T11SS: type 11 secretion systems
V_rms_: root mean square volts
vWA: von Willebrand factor type A
