# Supplementary material for: Bioinformatic discovery of type 11 secretion system (T11SS) cargo across the Proteobacteria
Source: Microb Genom. 2025 May 21;11(5):001406. doi: 10.1099/mgen.0.001406 (PMC12282271; doi:10.1099/mgen.0.001406)
Supplement: Uncited Supplementary Material 1. [file mgen-11-01406-s001.pdf]

Supplementary Figures

Bioinformatic discovery of type 11 secretion system (T11SS) cargo across the Proteobacteria

Authors: Alex S. Grossman[1,2], Nicholas C. Mucci[1], Sarah J. Kauffman[1], Jahirul Rafi[1], Heidi Goodrich-Blair[1,*]

[1]Department of Microbiology, University of Tennessee, Knoxville, Knoxville, TN 37996-0845

[2]Current address: The ADA Forsyth Institute, 245 First St, Cambridge, MA 02142

[*]To whom correspondence should be addressed: hgblair@utk.edu

Keywords: Co-occurrence; T11SS, Host-association; Cargo detection; Plasmin-sensitive protein; DUF1194.

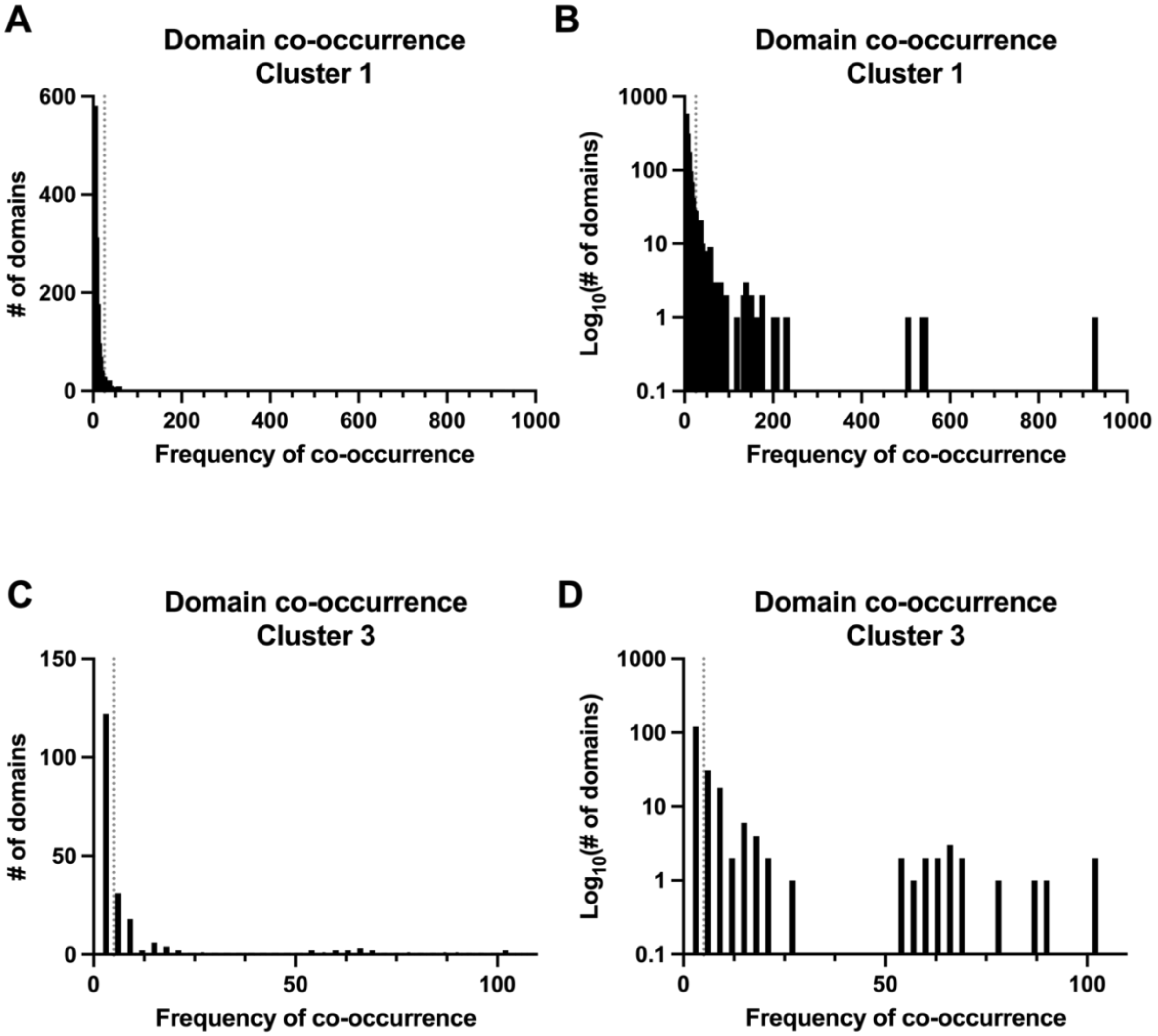

Supplemental Figure 1. **Histograms displaying the distribution of co-occurrence frequency amongst domains co-occurring with T11SS.** Genomic neighborhood analysis detects many domains within assayed loci (6 open reading frames up- and down-stream of each T11SS), however the majority of domains only co-occur a small number of times. To filter out rare or spurious co-occurrences, a threshold of co-occurrence is established relevant to the size of your dataset. Histograms depict the frequency of domain co-occurrence with animal-associated cluster 1 T11SS **(A)** and with marine-associated cluster 3 T11SS **(C)**. Log_{10} transformation of these values **(B, D)** helps to visualize domains with many co-occurrences. The dotted lines on all graphs show the thresholds chosen for filtering each dataset.

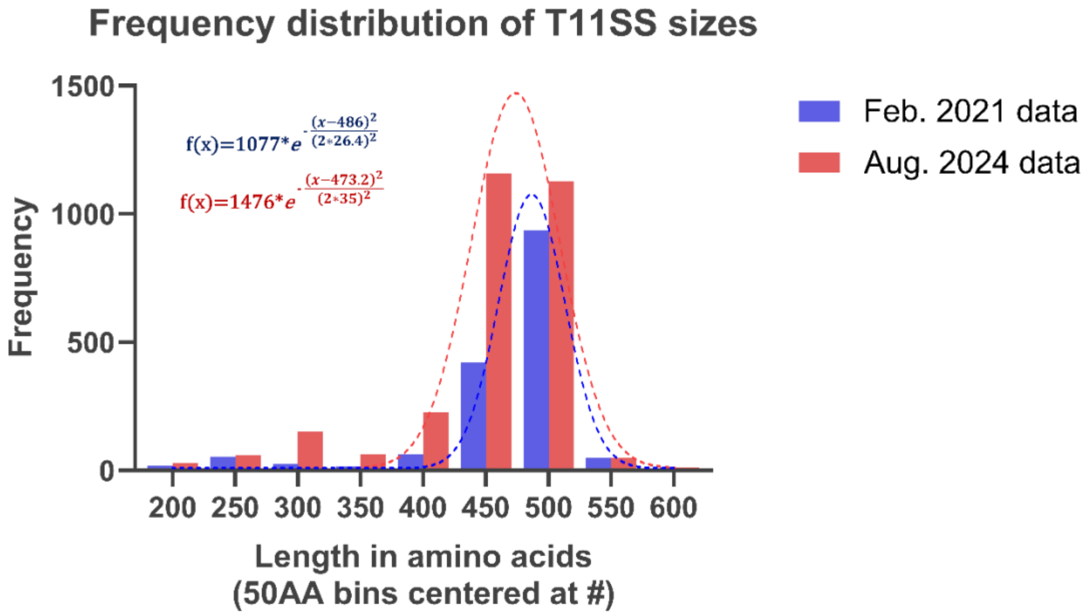

Supplemental Figure 2. **T11SS (DUF560) protein size distribution (in amino acids).** After examination of the size distribution, median was chosen as an appropriate measure of representative protein size over mean due to left-skewed distribution. The blue dataset was used to perform the T11SS co-occurrence analysis described in this manuscript. In the intervening time more homologs have been added to the Pfam database, the red dataset reflects these additions and their impact on the size distribution.

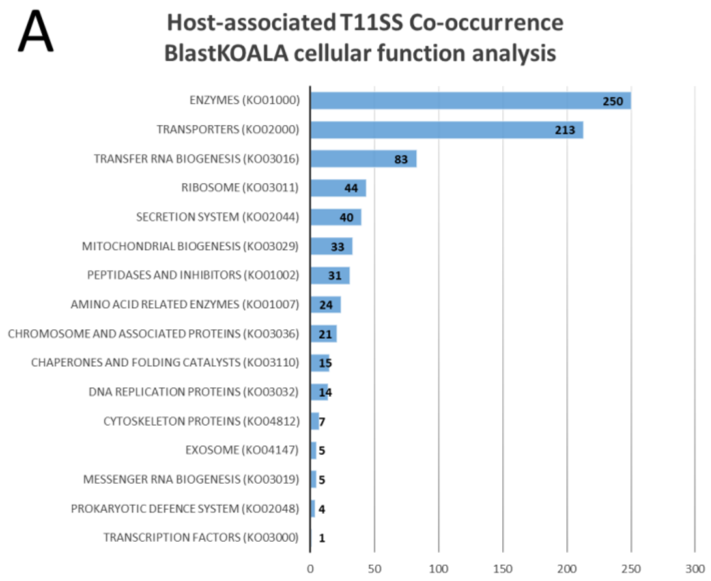

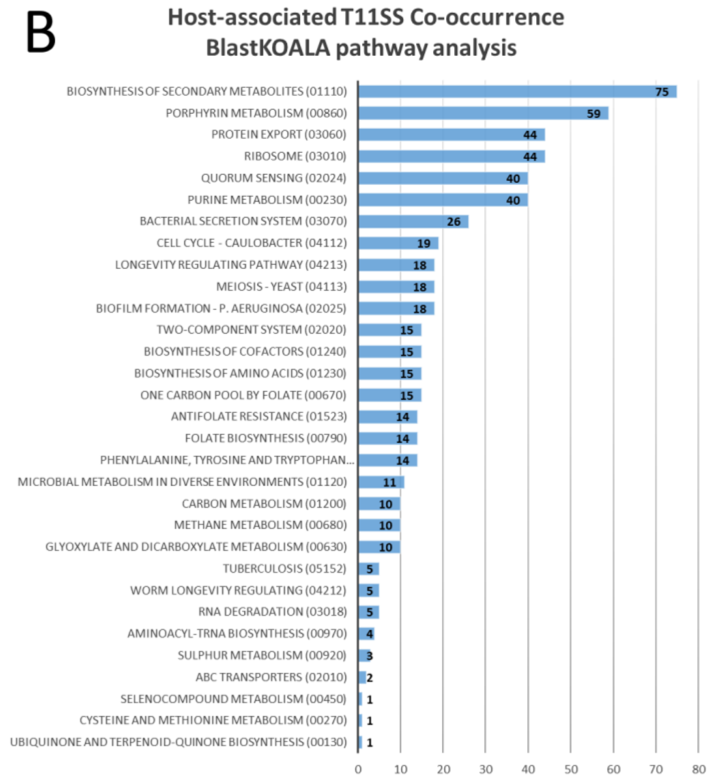

Supplemental Figure 3. **BlastKOALA supports Cluster 1 T11SS association with iron/heme uptake, protein export, and one-carbon metabolism**. BlastKOALA uses the BLAST alignment algorithm to assign functions to query sequences and reveal shared pathways. Cellular functions **(A)** were estimated using BRITE hierarchies and assigned, where possible, to known pathways **(B)** to detect commonalities.

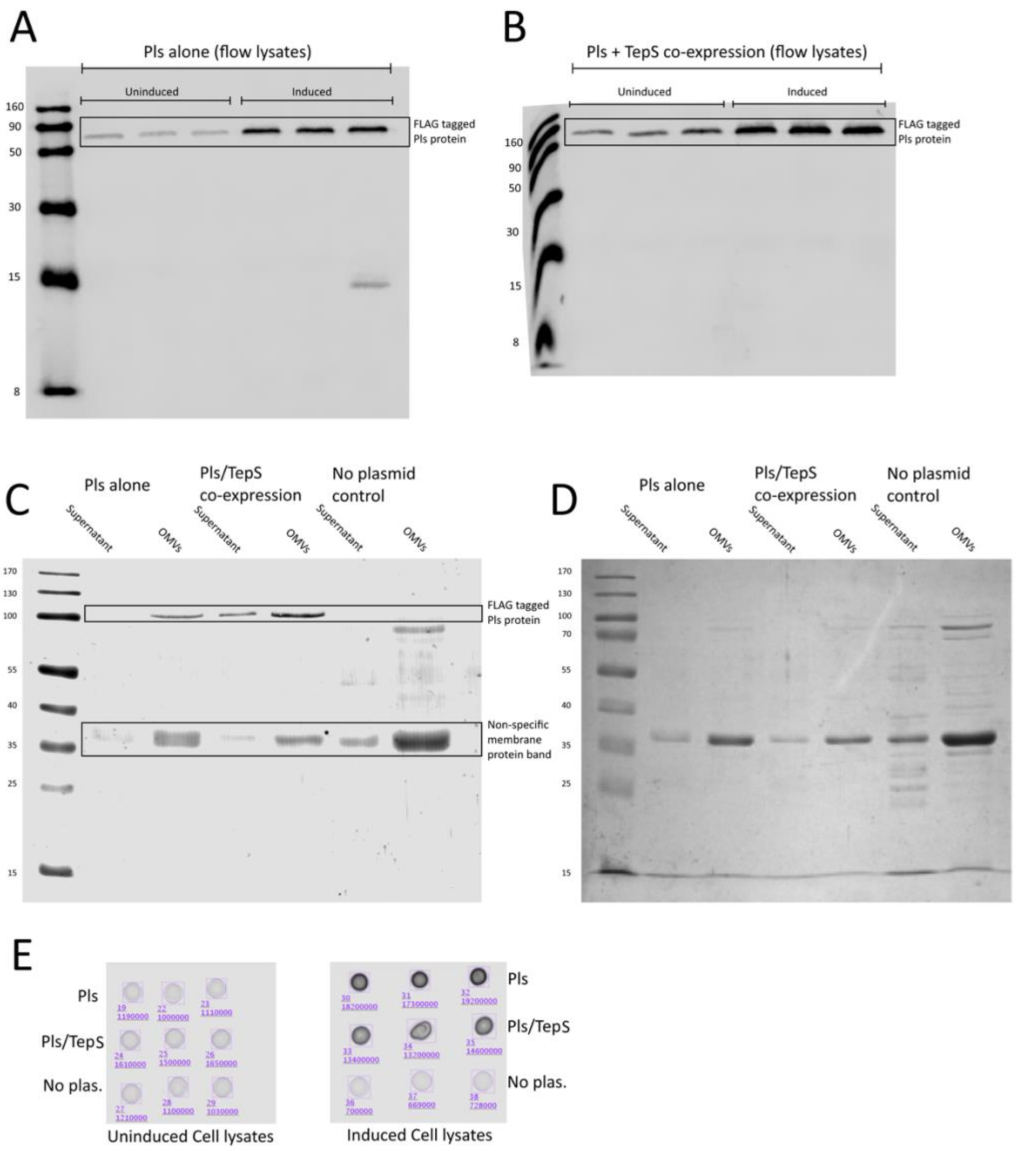

Supplemental Figure 4. **Localization of FLAG tagged Pls in the absence and presence of its cognate T11SS secretor.** Samples from *E. coli* cells expressing Pls-FLAG alone or Pls-FLAG and its cognate T11SS (TepS) were probed with α-FLAG antibody using Western blotting (lysates from flow cytometry samples, supernatants, and extracellular vesicles) **(panels A-C)** or dot blots (lysates from supernatant collections) **(panel E)** to determine if expression of the T11SS-protein TepS impacted Pls protein localization. Representative Western blots demonstrating comparable levels of Pls expression in flow cytometry cultures in Pls only **(A)** and Pls and TepS **(B)** expressing cells. **(C)** A representative Western blot showing α-FLAG-antibody reactive supernatant proteins separated into soluble and vesicle fractions. **(D)** A representative Coomassie stain showing total supernatant protein content separated into soluble and vesicle fractions. **(E)** Immuno-dot blots demonstrating comparable levels of Pls expression in lysates from supernatant collection cultures.

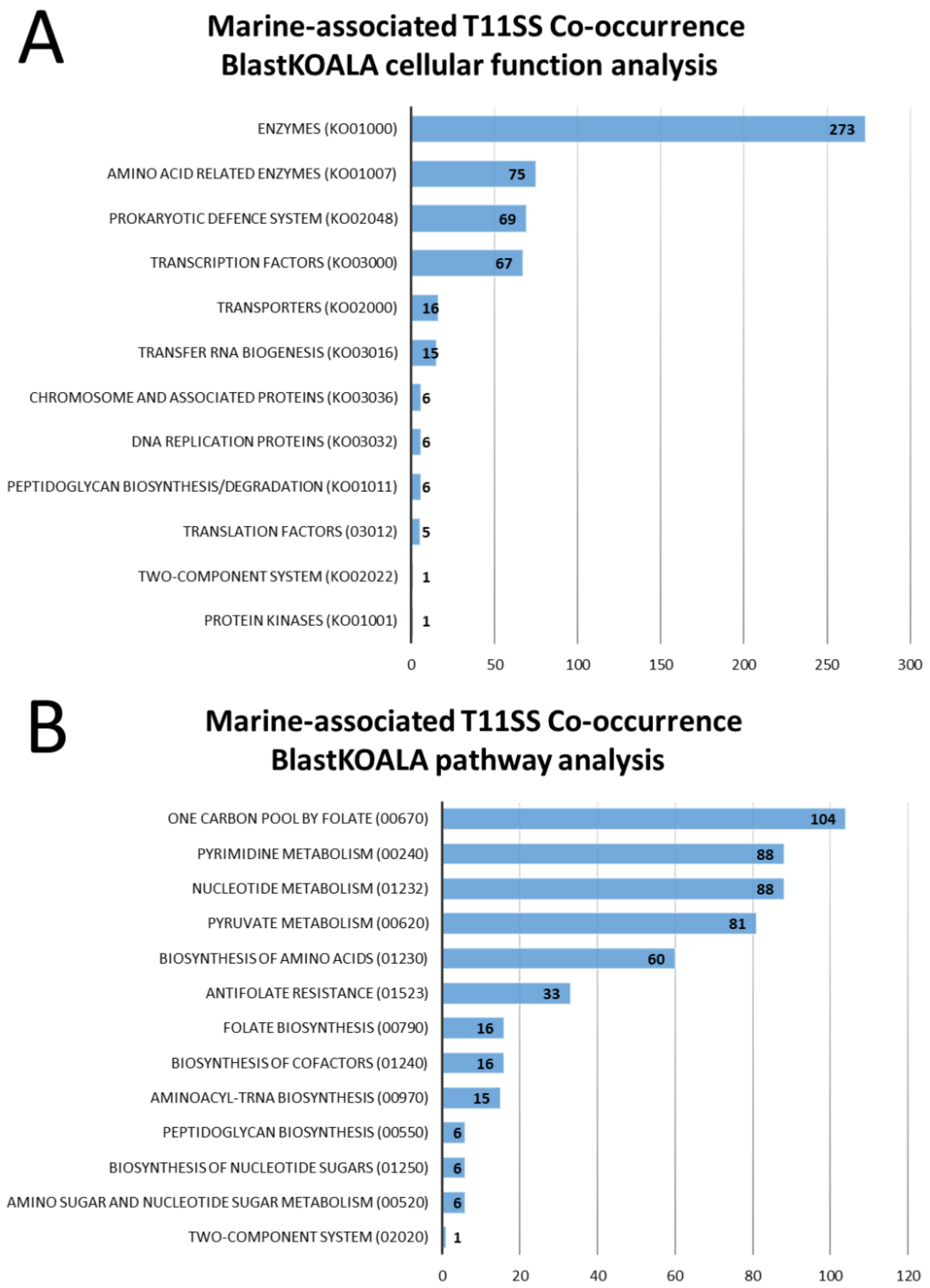

Supplemental Figure 5. **BlastKOALA supports Cluster 3 T11SS association with one-carbon metabolism, nucleotide metabolism, and the glyoxalase-detoxification pathway**. BlastKOALA uses the BLAST alignment algorithm to assign functions to query sequences and reveal shared pathways. Cellular functions **(A)** were estimated using BRITE hierarchies and assigned, where possible, to known pathways **(B)**.
